# Piezobiomimetic delivery nanosystem converts cold tumors to hot by parallel PANoptosis/STING activation in hepatocellular carcinoma

**DOI:** 10.1126/sciadv.aea6844

**Published:** 2026-06-17

**Authors:** Jiaoting E, Yuxuan Zhao, Xiaoling Zhang, Qiuqi Zhang, Ying Xu, Shanshan Liu, He Ding, Jiuxin Zhu, Piaoping Yang, Rui Xie

**Affiliations:** ^1^ Department of Digestive Internal Medicine, Harbin Medical University Cancer Hospital, Harbin 150081, P. R. China.; ^2^ Key Laboratory of Superlight Materials and Surface Technology, Ministry of Education, College of Materials Science and Chemical Engineering, Harbin Engineering University, Harbin 150001, P. R. China.; ^3^ Department of Pharmacology (State-Province Key Laboratories of Biomedicine Pharmaceutics of China, Key Laboratory of Cardiovascular Medicine Research, Ministry of Education), College of Pharmacy, Harbin Medical University, Harbin 150086, P. R. China.

## Abstract

Hepatocellular carcinoma (HCC) remains a challenge for therapeutic efficacy in converting cold tumors into hot ones. Herein, doping defect–engineered manganese perovskite piezoelectric nanocubes (Mn-BaZrO_3_, MBZO) integrated miltirone biomimetic delivery within tumor-derived nanovesicles are proposed for the parallel pyroptosis-apoptosis-necroptosis (PANoptosis) and the stimulator of interferon genes (STING) pathway activation to reshape the immune microenvironment, leading to tumor regression in HCC. Homologously targeting HCC cells, MBZO nanocubes accumulated and produced exogenous reactive oxygen species through the piezocatalytic effect. Specifically, miltirone accelerated oxidative stress amplification, potentially serving as an inducer of programmed cell death in HCC. Then, PANoptosis was activated to disrupt the cell barrier and release damage-associated molecular patterns, promoting immunogenicity. Concurrently, MBZO synergistically stimulated the STING pathway to evoke pro-inflammatory responses and elicit immunogenic cell death, triggering effector immune cell deployment (EICD). The piezobiomimetic delivery nanosystem bridged innate immunity activation and EICD to convert cold tumors into hot tumors and suppress tumor growth, thereby enhancing the efficacy of piezoimmunotherapy in HCC.

## Teaser

A biomimetic nanomedicine activates PANoptosis and STING pathways to enhance the efficacy of liver cancer management.

## INTRODUCTION

Liver cancer is one of the most prevalent malignancies and the third leading cause of cancer-related mortality globally (*1*, *2*). Hepatocellular carcinoma (HCC) contributed ~80% of the primary liver cancer burden (*3*). Despite substantial progress made in locoregional and systemic therapy, most patients with HCC are likely not to respond and ultimately succumb to their disease (*4*). Signatures of inflamed (hot) and noninflamed (cold) HCC have been associated with response to therapy. Unfortunately, most HCCs develop in a chronic immunosuppressive necroinflamed environment (*5*). The immunologically “cold” state tumor restricts cytolytic attack by tumor-infiltrating lymphocytes, resulting in a poor response to immunotherapy. Effector immune cell deployment (EICD), which encompasses the priming, circulation, activity, trafficking, and fate of antitumor effector immune cells, is a crucial step in transforming nonresponsive cold tumors into responsive hot ones (*6*). Consequently, there is an imperative need to investigate feasible EICD strategies to reprogram the immunosuppressive condition and convert cold tumors into hot ones by promoting immunogenicity, thereby enhancing therapeutic responses in HCC management.

Piezocatalytic therapy (PCT), a class of reactive oxygen species (ROS)–based therapeutic approaches that use piezoelectric nanomaterials for catalysis, has emerged as a promising protocol for reprogramming the immunosuppressive microenvironment and enhancing the immune response to tumors and as a prospective noninvasive antitumor approach due to its high spatiotemporal accuracy (*7*, *8*). The unique electron-releasing properties of piezocatalysts not only initiate a catalytic therapeutic modality but also enable the use of an external stimulus field with the advantages of safety, convenience, and controllable time and space (*9*, *10*). Among them, ultrasound (US), due to the inherent cavitation effect of piezomaterials, causes deformation of the surface of piezocatalysts, resulting in polarization and built-in electric fields in the material, allowing electrons and holes to separate and be attracted to opposite surfaces continuously (*11*, *12*). In aqueous solutions, surface charge carriers react with water or dissolved oxygen to produce ROS for cancer treatment (*13*). Such excessive production of ROS can effectively disrupt mitochondrial function by exacerbating oxidative stress, inducing programmed cell death (PCD), and causing the release of large amounts of damage-associated molecular patterns (DAMPs) to activate the antitumor immune response of the host (*7*, *14*, *15*). In addition, US irradiation exhibits a profound tissue penetration effect of up to 12 cm, a targeted focus on deep tumors, and a noninvasive nature, making piezoelectric material a potential candidate for treating tumors in deep tissue sites (*16*, *17*), including HCC.

The use of emerging modes of PCD to guide therapeutic strategies has been widely studied in cancer immunotherapy (*18*, *19*). Pyroptosis-apoptosis-necroptosis (PANoptosis), defined as an innate immune, lytic, and inflammatory PCD pathway, effectively inhibits cell growth and activates a potent immune response (*20*–*22*). Tumor-specific induction of PANoptosis generates a robust and long-lasting tumor-specific immune response by disrupting the cell barrier and causing dying tumor cells to release DAMPs (*23*, *24*). The integrated activation process, which encompasses key features of PANoptosis, cannot be fully recapitulated by any single form of PCD; therefore, it provides a rationale for developing combination therapies, particularly when monotherapies targeting individual molecules have proven ineffective (*22*, *25*, *26*). It is imperative to create a pragmatic approach to induce PANoptosis, which could have substantial implications for promoting the immunogenicity of HCC. The stimulator of interferon genes (STING) pathway plays a crucial role in immune responses, and it is highlighted as a potential therapeutic target for HCC (*27*, *28*). The manganese (Mn) ions serve as a stimulator of the STING pathway, even in the absence of double-stranded DNA, to activate antitumor immune responses (*29*–*31*). Activation of the STING pathway can promote interferon type I (IFN-I) secretion and the maturation of dendritic cells (DCs), enabling them to effectively present DAMPs and inflammatory substances released during PCD to CD8^+^ T cells, leading to immunogenic cell death (ICD) of tumor cells (*31*–*33*). Therefore, piezoimmunotherapy, which involves the parallel activation of the STING pathway and PCD pathways, could effectively enhance antitumor CD8^+^ T cell responses to restrain HCC progression.

**Fig. 1.**
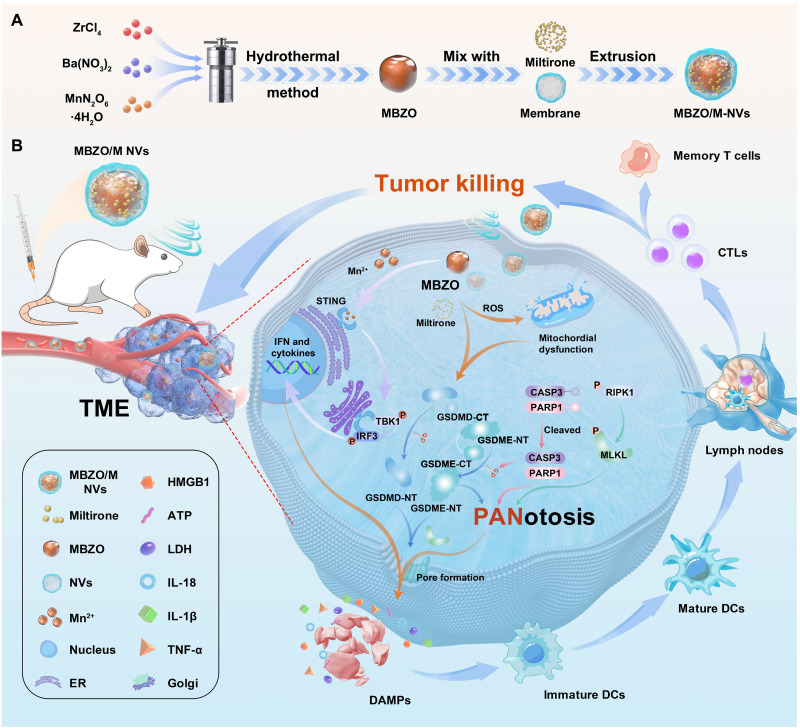
Scheme of the synthetic procedure and synergistic tumor piezoimmunotherapy of MBZO/M-NVs. (**A**) Preparation of MBZO/M-NVs. (**B**) MBZO/M-NVs enhance PCT efficacy and elicit robust antitumor immunity by activating PANoptosis and the STING pathway.

Herein, the delivery nanosystem, comprising Huh7-derived nanovesicles (NVs) coating miltirone (M) and doping defect–engineered manganese perovskite piezoelectric nanocubes (Mn-BaZrO_3_, MBZO), finally determined as MBZO/M-NVs, was proposed to mitigate the immunosuppressive condition and convert cold tumors into hot ones by parallel PANoptosis/STING pathway activation (Fig. 1). Through the homologous targeting property of Huh7-NVs, the piezobiomimetic delivery nanosystem preferentially entered and accumulated in hepatoma cells via receptor-mediated endocytosis. Piezoelectric MBZO catalyzed oxidation reactions through inherent piezoelectric properties, whereas miltirone accelerated endogenous ROS generation and rapidly disrupted mitochondrial function, reshaping the immunologically “cold” properties of the HCC tumor microenvironment (TME). The proliferation of ROS, accumulation of MBZO, and release of PCD inducer miltirone synergistically activate PANoptosis. Notably, PANoptosis induced by the biomimetic delivery nanosystem not only inhibited tumor growth but also promoted the release of DAMPs. Meanwhile, intracellular MBZO accumulation potentiated STING pathway activation and interferon-stimulated gene (ISG) expression, further triggering EICD, which not only reinvigorates CD8^+^ T cells within the TME but also licenses DCs for CD8^+^ T cell priming and clonal expansion, as well as activating natural killer (NK) cells to kill tumor cells. Overall, this piezobiomimetic delivery nanosystem offers a feasible EICD strategy to convert cold tumors into hot ones, leading to tumor regression by simultaneously stimulating PANoptosis and the STING pathway, thereby redefining piezoimmunotherapy against HCC.

## RESULTS

### Synthesis and characterization of piezonanocubes

**Fig. 2.**
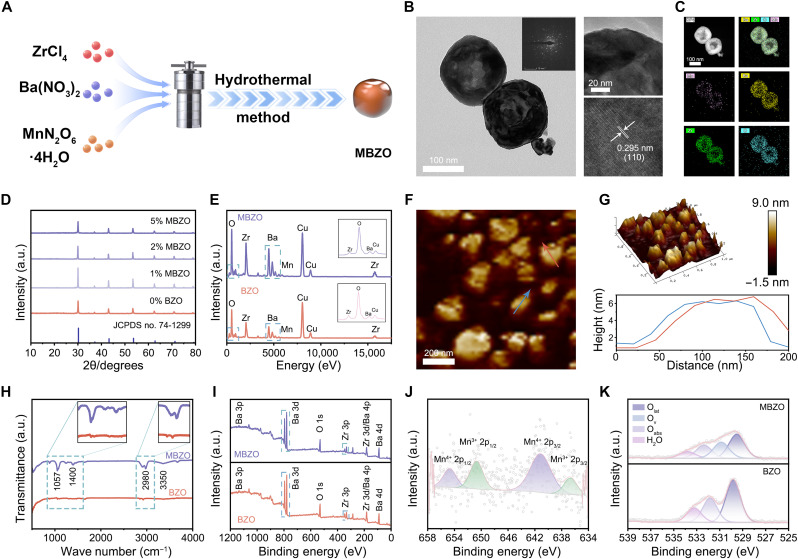
Synthesis procedure and characterizations of piezonanocubes. (**A**) Schematic illustration of the synthetic process of MBZO nanocubes. (**B**) TEM images of 5% MBZO nanocubes and high-resolution TEM images of the enlarged lattice images. (**C**) HAADF-STEM and the corresponding area elemental mapping images. (**D**) XRD spectra of BZO, 1% MBZO, 2% MBZO, and 5% MBZO. a.u., arbitrary units. (**E**) EDX spectrum of BZO and 5% MBZO nanocubes. (**F**) 2D AFM image and (**G**) 3D visualizations of the current signal distribution obtained through electrical AFM, alongside corresponding height profiles of the MBZO nanocubes. (**H**) FTIR spectra of BZO and MBZO nanocubes. (**I**) Survey XPS spectrum, (**J**) high-resolution Mn 2p spectrum, and (**K**) high-resolution O 1s spectrum of BZO and MBZO nanocubes.

In a pursuit to construct a promising protocol for boosting the immune response to cancer and a prospective noninvasive antitumor approach through PCT, the hollow intelligent therapeutic sonopiezoelectric material MBZO nanocubes rich in oxygen vacancies (O_V_s) was elaborately designed, which hold great potential to trigger cellular oxidative stress for effective treatment, and synthesized by a hydrothermal method (Fig. 2A). The Mn element was doped in situ on the barium (Ba) site, variable valence metal ions were introduced, the electronic structure of the material was adjusted, and the energy gap (*E*_g_) was reduced. At the same time, rich O_V_s were formed as electron traps, improving the lifetime of piezoelectric charge carriers and thus facilitating charge separation. The hollow structure of the nanocubes increased the specific surface area, thereby enhancing their catalytic activity. By adjusting the reagent feed ratio, undoped BaZrO_3_ (BZO) nanocubes and MBZO nanocubes doped with three different Mn ratios were prepared, with theoretical doping ratios of 1, 2, and 5%, respectively. According to the large field transmission electron microscopy (TEM) images, the 5% MBZO nanocubes exhibited a spherical structural feature with an average diameter of 153.3 ± 43.0 nm. In contrast, the average diameter of the BZO nanocubes was 139.1 ± 29.9 nm. Similarly, the particle sizes of 1% MBZO and 2% MBZO were 142.3 ± 55.2 and 148.9 ± 75.4 nm, respectively, which showed a slight increase with an increase in Mn doping ratio (fig. S1). The differences may be due to the introduction of O_V_s by Mn doping, which leads to lattice expansion and an increase in lattice spacing, thereby increasing the particle size. High-resolution transmission electron microscopy (HRTEM) images confirmed the lattice plane spacing of 0.295 nm, corresponding to the (110) atomic plane of the face-centered cubic phase of 5% MBZO (Fig. 2B). The lattice spacing of the same (110) plane was 0.288, 0.292, and 0.294 nm for BZO, 1% MBZO, and 2% MBZO, respectively, which confirmed the phenomenon of lattice expansion (fig. S2). Moreover, the high-angle annular dark-field scanning transmission electron microscopy (HAAD-STEM) element mapping images confirmed the distribution of Mn, Ba, zirconium (Zr), and oxygen (O) elements on the shell of 5% MBZO nanocubes, revealing a clear hollow structure at the center (Fig. 2C). Similarly, the morphology images and element distribution demonstrate that all samples had structural features similar to 5% MBZO nanocubes (figs. S2 and S3). Powder x-ray diffraction (XRD) patterns showed that synthesized BZO nanocubes have three prominent diffraction peaks at 30.20°, 43.24°, and 53.64°, which corresponded to the (110), (200), and (211) crystal planes of the standard face-centered cubic phase structure (JCPDS no. 74-1299) (Fig. 2D). No additional peak indicative of the manganese oxide was observed in 1, 2, and 5% MBZO nanocubes, demonstrating that the Mn element entered the lattice of MBZO nanocubes. The diffraction peaks of 1, 2, and 5% MBZO nanocubes exhibited a slight shift to lower angles, indicating that the doping of Mn would lead to lattice expansion and an enlarged *d*-spacing, consistent with the HRTEM images. The proportions of Mn, Ba, and Zr were measured using inductively coupled plasma mass spectrometry (ICP-MS) to determine the content of corresponding elements in the samples. The actual Mn content ratios of 1, 2, and 5% MBZO nanocubes were about 1.68, 2.01, and 5.14%, respectively. In contrast, the BZO nanocubes contain no Mn element, which proves that the actual doping ratio of the nanocubes has no notable difference from the theoretical doping ratio (table S1). The energy-dispersive spectroscopy (EDX) spectrum also confirmed the conclusion (Fig. 2E and fig. S4). On the basis of the above data, different proportions of Mn doping alter the lattice parameters, with a positive feedback relationship between the amount of O_V_s and the *d*-spacing of the nanocubes. At the same time, 5% MBZO nanocubes had the highest O_V_s ratio, and the introduction of more Mn elements represented an improvement in the ability to change ion valence states, which is more likely to trigger Fenton-like reactions. Therefore, 5% MBZO nanocubes with the optimal crystal structure were selected for subsequent experiments.

Next, the atomic force microscopy (AFM) images and the corresponding height profiles revealed that the MBZO nanocube exhibited a cubic spherical feature in terms of height (Fig. 2, F and G). Meanwhile, Fourier transform infrared (FTIR) spectroscopy characterizes the MBZO nanocubes exhibit multiple distinct absorption bands at 1057, 1400, 2980, and 3350 cm^−1^, respectively (Fig. 2H). The presence of hydroxyl radical (·OH) groups was attributed to the doping of Mn, which introduces O_V_s on the surface of MBZO nanocubes, adsorbs hydrogen impurities, and then combines with lattice oxygen to generate ·OH groups (*34*–*36*). The absorption band at 1400 cm^−1^ represents Ba─O stretching, whereas the absorption bands at 1057, 2980, and 3350 cm^−1^ can be attributed to the bending or stretching vibration of the hydroxyl groups (*37*–*39*). The above data characterize the basic crystalline cell structures and surface vacancy structures of BZO nanocubes and MBZO nanocubes. To further illustrate the effect of Mn doping on the surface composition and chemical state of MBZO nanocubes, x-ray photoelectron spectroscopy (XPS) characterization was performed. The survey XPS spectrum demonstrates that Mn, Ba, Zr, and O elements simultaneously exist in MBZO nanocubes (Fig. 2I). The high-resolution spectra of Mn 2p display four peaks, including the peaks at 650.69 eV (Mn 2p_1/2_) and 636.68 eV (Mn 2p_3/2_), which are attributed to Mn^3+^. In contrast, the peaks at 654.71 eV (Mn 2p_1/2_) and 641.14 eV (Mn 2p_3/2_) are ascribed to Mn^4+^ (Fig. 2J) (*40*). The presence of Mn ions in different valence states enhances the catalytic ability and the activity of Fenton-like reactions.

Meanwhile, the high-resolution spectra of Ba 3d and Zr 3d show only one valence state, proving that the occurrence of Fenton-like reactions was not related to these two elements (fig. S5). The high-resolution spectrum of O 1s reveals that Mn doping induces a significant blue shift in the spectrum and imports a peak that represents the O_V_ (532.37 eV) (Fig. 2K) (*36*, *41*). According to the peak area, the proportion of the O_V_ in the MBZO crystal is ~21.89%. In summary, the prepared MBZO nanocubes exhibit a uniform nanoscale hollow spherical structure, abundant O_V_s, and variable valence metal ions. The face-centered cubic lattice allows for the presence of multiple different octahedral structural units within a crystal cell, which cooperate with O_V_s as electron traps to promote the long-term separation of charge carriers. The hollow structure increases the specific surface area, exposing catalytic sites. Then, the particle size of around 150 nm ensures that the volume of MBZO nanocubes allows inherent piezoelectric properties to be fully used, thereby promising potential in catalytic applications.

### Density functional theory for the piezocatalytic mechanism

**Fig. 3.**
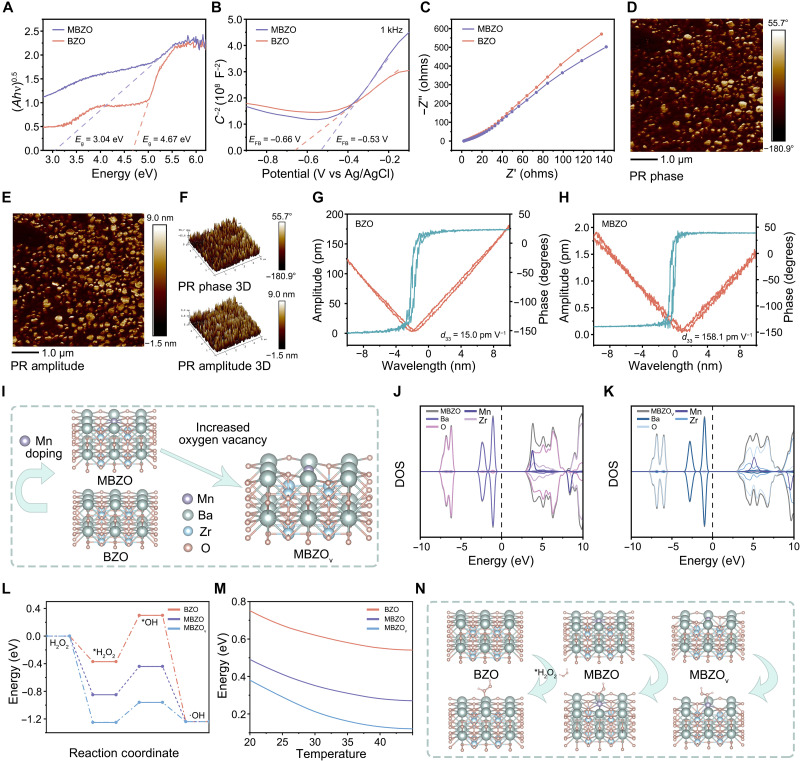
Characterization of the piezocatalytic and DFT. (**A**) UV-vis energy bandgap of BZO and MBZO nanocubes. (**B**) Mott-Schottky plots of BZO and MBZO nanocubes. (**C**) EIS spectra of BZO and MBZO nanocubes with continuous US. (**D** to **F**) PFM morphology of MBZO nanocubes in (D) the phase image, (E) the amplitude image, and (F) the corresponding 3D views. (**G** and **H**) PFM phase hysteresis loop and amplitude butterfly loop of (G) BZO and (H) MBZO. (**I**) Schematic illustration of the BZO and MBZO models. (**J** and **K**) DOS profiles of (J) MBZO and (K) MBZO_v_ nanocubes. (**L**) Energy diagrams of the catalytic pathways of the piezocatalytic processes of simulated BZO, MBZO, and MBZO_v_ nanocubes. (**M**) Energy diagrams of the catalytic pathways of the PCT processes of simulated nanocubes with temperature variation. (**N**) Modeling surface structure of defect-free BZO and defect-rich MBZO in PCT processes.

Doping elements can effectively reduce the bandgap of the nanomaterials (*35*, *42*, *43*). Therefore, the energy band structures of BZO and MBZO nanocubes were compared using an ultraviolet-visible (UV-vis) diffuse reflection spectrum. By analyzing the obtained data, the bandgaps of BZO and MBZO nanocubes are calculated to be 4.67 and 3.04 eV, respectively, proving that Mn doping effectively reduces the difficulty of charge carrier transitions in MBZO nanocubes excited by US (Fig. 3A), which may be due to the introduction of additional energy levels in the bandgap by doping elements. At the same time, the Mott-Schottky relationships of BZO and MBZO nanocubes exhibit positive slopes indicative of n-type conductivity (Fig. 3B). The flat band potentials (*E*_FB_) of BZO and MBZO nanocubes are determined to be −0.66 and −0.53 V (versus Ag/AgCl), respectively. Therefore, the *E*_FB_ versus the standard hydrogen electrode (SHE) can be calculated to be −0.05 and 0.08 V, respectively. For a typical n-type semiconductor, the *E*_FB_ value obtained from the Mott-Schottky plot is usually 0.1 V higher than the conduction band minimum (CBM) (*44*). Thus, the CBM of BZO and MBZO nanocubes could be estimated to be −0.15 and −0.02 V (versus NHE), respectively. According to the data above, the valence band minimum (VBM) was calculated to be 4.52 and 3.02 V for BZO and MBZO nanocubes, respectively, which was conducive to the occurrence of water-based reduction reactions. Furthermore, electrochemical impedance spectroscopy (EIS) shows that the arc radius of MBZO nanocubes in the EIS Nyquist plot significantly reduced, indicating a decrease in charge carrier migration resistance and an improvement in migration behavior (Fig. 3C). The results demonstrates Mn doping can regulate the electronic structure of MBZO nanocubes, reduce the bandgap, and promote electronic transitions on the conduction band (CB) and valence band (VB).

In addition to the electronic structure adjustment caused by ion doping, the inherent piezoelectric properties of MBZO nanocubes also play a crucial role in its catalytic performance. Under piezoresponse force microscopy (PFM), the phase (Fig. 3D), amplitude (Fig. 3E) images, and the representative three-dimensional (3D) views (Fig. 3F) exhibit significant phase differences. The results demonstrate that the MBZO nanocubes have distinct piezoelectricity. In addition, BZO and MBZO nanocubes exhibit a representative butterfly amplitude graph and an average phase contrast of nearly 180°, confirming that the applied electric field causes strain in the crystal structures. It has been noted that the MBZO nanocubes exhibited a 10 times higher piezoelectric coefficient (*d*_33_) of 158.1 pm V^−1^ in comparison to the BZO nanocubes (15.0 pm V^−1^) (Fig. 3, G and H). The high piezoelectric performance resulted in strong charge carrier separation, and the band tilt caused by the separated charges will effectively enhance the catalytic performance (*45*). Therefore, the MBZO nanocubes have meaningful structural advantages in sonocatalysis properties.

The electronic structures and PCT mechanisms of MBZO nanocubes were further investigated by density functional theory (DFT) calculations using the Vienna ab initio simulation package. The crystal structures of BZO, MBZO, and MBZO increasing oxygen vacancy (MBZO_V_) nanocubes are illustrated (Fig. 3I). According to the formation energy calculation of different replacement sites, Mn doping sites have been confirmed to be on the Ba sites (fig. S6). The similarity in valence states and electronic structures between Mn and Ba atoms contributes to this phenomenon. COMSOL simulation results indicate that the electric field generated by the MBZO nanocubes is more pronounced than that of the BZO nanocubes under ultrasonic activation, which can be attributed to the strong piezoelectric properties of the MBZO nanocubes (fig. S7). In addition, the total density of states (DOS) for Mn, Ba, Zr, and O atoms is also calculated (Fig. 3, J and K). The Ba state and O state primarily dominate the VBM of MBZO, whereas the CBM of MBZO is dominated mainly by the Zr state, with a small contribution from the Mn state. The results indicate that holes migrate to the (BaO_6_) octahedral layer, whereas electrons migrate to the (ZrO_6_) octahedral layer (*46*). Owing to the aggregation of electrons and holes in different structural domains, the spatial separation of charge carriers is achieved in a single cell of the MBZO nanocubes. Furthermore, the O_V_s introduced by Mn doping have no significant alteration in the total DOS structure. The energy barrier required to excite electronic transitions is slightly reduced with the existence of O_V_s (from 3.02 to 2.99 eV), confirming the necessity of O_V_s in the electronic structure of MBZO_V_. In addition, O_V_s of MBZO could serve as electron traps that receive electrons from CB, and as an obstacle to the electron movement process, effectively reducing the recombination efficiency of the charge carriers.

To reveal the reaction mechanism of MBZO nanocubes, the catalytic processes of hydrogen peroxide (H_2_O_2_) were investigated comprehensively using optimized surfaces. The adsorption energy of H_2_O_2_ on different surface sites was calculated. The result shows the maximum negative value at the O_V_s sites on the surface, which is beneficial for enriching reactants and enhancing the efficiency of the piezocatalytic process (fig. S8). The corresponding free energy diagram of catalytic stages is detected at 25°C (Fig. 3L). The first step involves the surface activation and dissociation of H_2_O_2_, a thermodynamically favorable process. In the second step, H_2_O_2_ would be decomposed into ·OH, which requires overcoming an energy barrier and thus becomes the rate-determining step. The O_V_s on the surface of the MBZO_V_ nanocubes provide the bottommost free energy throughout the entire reaction process, thereby promoting the generation of ·OH. According to the free energy diagram at different temperatures, increasing the temperature can facilitate H_2_O_2_ adsorption and reduce the energy barrier for ·OH formation (Fig. 3, M and N). Thus, characterizing the catalytic and sonothermal properties of MBZO nanocubes is necessary for verifying the therapeutic mechanism.

### Piezoelectric catalysis and sonothermal performance

**Fig. 4.**
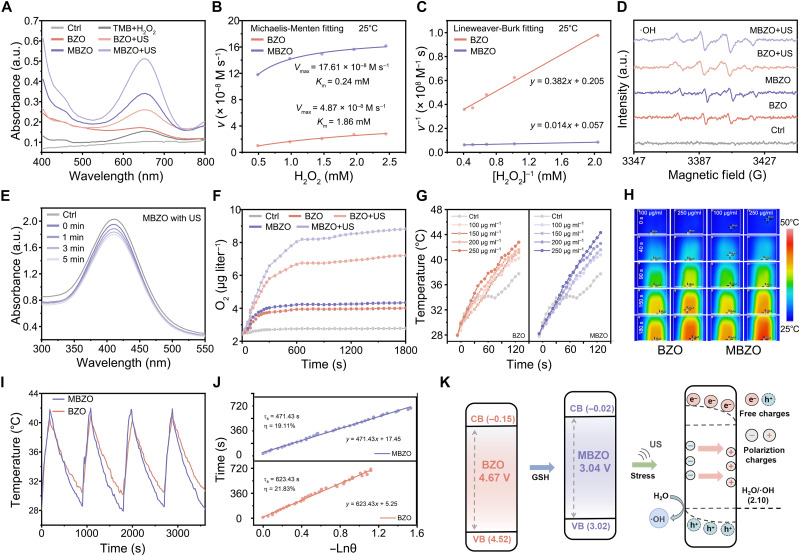
Characterization of piezoelectric catalysis and sonothermal effects. (**A**) UV-vis absorption spectra of oxTMB. (**B**) Michaelis-Menten fitting and (**C**) Lineweaver-Burk fitting BZO and MBZO with H_2_O_2_ as a substrate. (**D**) ESR spectra of ·OH production. (**E**) UV-vis absorption spectra of DTNB in MBZO nanocubes solutions with US for different reaction times. (**F**) O_2_ generation profiles. (**G**) Sonothermal heating curves of dispersed BZO and MBZO nanocubes with different concentrations. (**H**) Infrared thermal imaging of BZO and MBZO nanocube solutions with concentrations of 100 and 250 μg ml^−1^ with US irradiation (power density: 1.0 W cm^−2^). (**I**) Sonothermal stability performance of BZO and MBZO nanocubes solution over four on/off cycles with continuous US irradiation. (**J**) Relationship between −Lnθ and the time. (**K**) Schematic diagram of the intrinsic energy bands of piezoelectric nanocubes and the tilted energy bands favoring ·OH production under ultrasonically excited piezoelectric fields.

On the basis of the energy band structure characteristics of the MBZO nanocubes, the properties of ROS generation were verified. The characteristic absorbance peaks of oxidized 3,3′,5,5′-tetramethylbenzidine (oxTMB) appear at 652 nm by adding BZO and MBZO nanocubes, thus suggesting the nanocubes can induce the decomposition of H_2_O_2_ to generate ·OH (Fig. 4A). The absorption peak of MBZO nanocubes is significantly higher than that of BZO, indicating that the introduction of O_V_s improved the catalytic efficiency. In addition, ultrasonic exposure can also considerably enhance the generation of ·OH, confirming the synergistic effect between the strong piezoelectric effect and the ultrasonic cavitation effect of MBZO nanocubes. The absorbance peaks were also detected under different reaction times and pH conditions using TMB (figs. S9 and S10). The results indicate the promotion of ·OH generation with increasing time and decreasing pH, which verifies that the MBZO nanocubes have an adaptive response for the acidic TME.

The initial reaction rate of steady-state kinetics analysis was determined using the Beer-Lambert law, and the Michael-Menten saturation curve was used for fitting (Fig. 4, B and C, and fig. S11). The catalytic maximum rates (*V*_m_) of BZO and MBZO nanocubes are 4.87 × 10^−8^ and 17.61 × 10^−8^ M s^−1^, and the concentrations of substrate at half the maximum rate (*K*_m_) are 1.86 and 0.24 mM, respectively. *V*_m_ represents the catalytic rate of nanomaterials, and *K*_m_ represents the affinity of nanomaterials to the substrate. The catalytic efficiency of MBZO nanocubes is significantly higher than that of BZO, indicating that MBZO nanocubes have superiority in structure and composition. The electron spin resonance (ESR) spectrum displays an integral intensity of 1:2:2:1, which grouping trend is consistent with the results of the TMB color reaction, qualitatively characterizing the effects of Mn doping and US exposure (Fig. 4D). In addition, the piezocatalytic activities of BZO and MBZO nanocubes were characterized by 5,5′-dithiobis(2-nitrobenzoic acid) (DTNB) probe and the dissolved oxygen meter, respectively (Fig. 4E and fig. S12). The fading of the DTNB probe confirms the consumption of GSH [glutathione (reduced form)], which disrupts the redox balance and enhances the therapeutic effect of ROS. The production of oxygen (O_2_) alleviates tumor hypoxia, resulting in the inhibition of tumor proliferation.

Next, the heating curves of different concentrations of BZO and MBZO nanocubes at various power densities were characterized under US conditions (Fig. 4, F, G, and H). The heating curves of BZO and MBZO nanocubes exhibit a linear increase with increasing time under ultrasonic exposure. Under the conditions of a concentration of 100 μg ml^−1^ and a power density of 1.0 W cm^−2^, the solution could be heated from room temperature to 42.1°C within 180 s, meeting the requirement of mild thermal therapy. Concentration-dependent and power density–dependent sonothermal effects are observed, which facilitate the regulation of the temperature gradient by adjusting the injected dose and the power density of the US (fig. S13). Four heating and cooling cycles indicate that both BZO and MBZO nanocubes have the heating stability that meets the therapeutic requirements (Fig. 4I). According to the fitting of a single heating and cooling cycle, the sonothermal conversion efficiencies (η) of BZO and MBZO nanocubes are calculated to be 19.11 and 21.83%, respectively (Fig. 4J). The results demonstrate that the enhanced sonothermal effect also plays a crucial role in PCT caused by MBZO nanocubes. Last, a schematic illustration of electronic energy bands is constructed to conclude the piezoelectric properties of nanocubes (Fig. 4K). For the MBZO nanocubes, the CBM value is more favorable than the O_2_/O^2−^ and ^1^O_2_ potential (−0.33 V), making ·O^2−^ and ^1^O_2_ generation energetically unfavorable. However, the VBM value of MBZO is beneficial to the H_2_O/·OH (2.10 V) redox reaction, theoretically capable of ·OH generation. For BZO nanocubes, the CBM value is also unsuitable for ·O^2−^ generation, whereas the VBM is more positive than that of MBZO nanocubes, theoretically indicating favorable ·OH generation than MBZO. Nonetheless, MBZO nanocubes have a narrower bandgap, better electronic structure, and higher piezoelectric properties, and the doping of Mn introduces additional O_V_s on the surface, which can capture transition electrons, enhancing the long-term charge carrier separation. Both PCT and sonothermal therapy are efficient strategies under US irradiation. Accordingly, piezoelectric nanocubes achieve outstanding therapeutic effects through synergistic piezoelectric catalysis and sonothermal effects.

### In vitro cytotoxicity evaluation

**Fig. 5.**
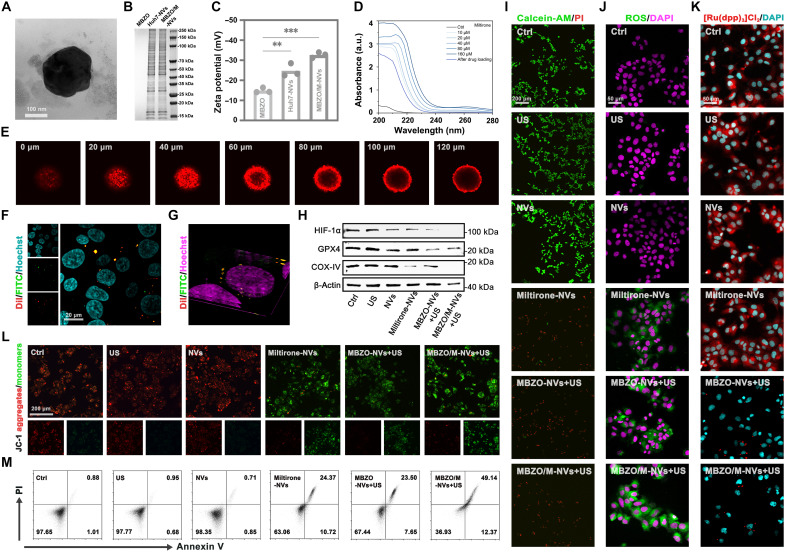
In vitro antitumor performance of PCT. (**A**) TEM image of MBZO/M-NVs. (**B** and **C**) SDS-PAGE result (B) and zeta potential (C) of MBZO, Huh7-NVs, and MBZO/M-NVs. (**D**) UV-vis absorption spectra of the miltirone. (**E**) CLSM images of Huh7 multicellular spheroids cocultured with DiI-labeled MBZO/M-NVs. (**F**) Intracellular location of FITC-labeled MBZO nanocubes and DiI-labeled Huh7-NVs and (**G**) 3D visualizations in Huh7 cells. (**H**) Western blot detected the TME-related proteins. CLSM images of (**I**) calcein-AM/PI double staining, (**J**) intracellular ROS level, (**K**) intracellular O_2_ production, and (**L**) JC-1 staining. (**M**) Flow cytometry analysis of the apoptosis in Huh7 cells. Data are presented as means ± SD (*n* = 3). Statistical analysis was determined using one-way ANOVA followed by Tukey’s post hoc test (Tukey’s post hoc test) (**P < 0.01; ***P < 0.001).

Huh7-NVs serving as a biomimetic homologous targeting nanocarrier were used to encapsulate the HCC pyroptosis inducer miltirone (*47*), followed by loading with piezoelectric MBZO nanocubes. TEM and HAADF-STEM were used to reveal the morphology and elemental mapping of MBZO/M-NVs. The TEM image shows the typical core-shell structure, a distinct circular membrane (shell) covering the MBZO nanocubes (core) (Fig. 5A), and the elemental mappings confirm the element distribution of MBZO nanocube and lipid bilayer (fig. S14A). Biological characterization via SDS–polyacrylamide gel electrophoresis (SDS-PAGE) with Coomassie blue staining reveals a diverse range of membrane-associated proteins in MBZO/M-NVs and Huh7-NVs. Furthermore, under identical protein loading conditions, no significant difference in protein expression was observed between the MBZO/M-NVs and Huh7-NVs groups, indicating minimal functional loss of NVs during the encapsulation process (Fig. 5B). Meanwhile, surface charge validation demonstrates enhanced electronegativity of MBZO/M-NVs (−32.63 ± 1.36 mV) compared to MBZO nanocubes (−14.90 ± 1.30 mV), improving the stability of blood circulation and achieving optimal affinity for the TME, via binding with negatively charged Huh7-NVs (−24.77 ± 3.26 mV) (Fig. 5C). Miltirone, as an inducer of PCD in HCC cell lines, was loaded into Huh7-NVs. To determine the loading efficiency, the UV-vis absorption spectra of different concentrations of miltirone and the rest of miltirone in the supernatant after loading were detected (Fig. 5D). The concentration of unloaded miltirone was calculated on the basis of a standard curve of concentration and absorbance (fig. S14B). The absorbance peak at 212 nm detected the loading efficiency of miltirone more than 90% in the MBZO/M-NVs group. The tumor penetration capability was validated in Huh7 multicellular spheroids. Confocal laser scanning microscopy (CLSM) *z*-stack imaging reveals the progressive penetration of 1,1′-dioctadecyl-3,3,3′,3′-tetramethylindocarbocyanine perchlorate (DiI)–labeled nanocubes at a scanning depth of 60 to 80 μm (Fig. 5E). Next, the integration efficacy of Huh7-NVs and MBZO nanocubes was confirmed when dual-fluorescence tracking detected spatial colocalization of fluorescein isothiocyanate (FITC)–labeled MBZO nanocubes and DiI-stained MBZO/M-NVs in overcoming biological barriers through homologous targeting (Fig. 5, F and G), establishing the biomimetic delivery system as an applicable nanoplatform for precision PCT.

The cytotoxicity of treatments with varying concentrations (0 to 100 μg ml^−1^) was assessed on Huh7 cells using the Cell Counting Kit-8 (CCK-8) assay to evaluate the bioeffects after the internalization of nanoparticles. In contrast to the negligible cytotoxicity of US and the weak toxic effect of the treatment with miltirone-NVs or MBZO-NVs+US, the MBZO/M-NVs+US group demonstrates dose-dependent and time-dependent cytotoxicity, achieving an effective inhibition rate at 100 μg ml^−1^, up to 86.85% (fig. S15, A and B). To investigate the biosafety and specificity of MBZO/M-NVs toward HCC cell lines, the human umbilical cord endothelial cells (HUVECs) and C57BL/6N mouse-derived myocardial fibroblasts were treated with MBZO/M-NVs+US, and then the cell viability was assessed using CCK-8 assays. The results show that there is no obvious cytotoxicity to HUVECs and fibroblasts with MBZO/M-NVs (100 μg ml^−1^) under US irradiation, indirectly supporting the biological safety within a certain range and homologous targeting capability of the nanosystem (fig. S15, C and D). Concurrently, the outcomes also validate the biosafety profile of US irradiation and NVs, demonstrating that NV encapsulation and US irradiation exert an insignificant oncogenic effect on tumor cell proliferation. It also hints that the enhanced therapeutic efficacy likely stems from the synergistic effect of PCT by MBZO with US irradiation and the curative effect of miltirone.

Considering the intuitional impacts of the tumor cell damage during the therapeutic process, the TME metabolism-associated protein expression profiles, including hypoxia-inducible factor 1α (HIF-1α), cytochrome c oxidase subunit IV (COX IV), and glutathione peroxidase 4 (GPX4), were preliminarily analyzed. The primary molecular mechanisms induced by hypoxia are mediated by the HIF, promoting immune escape through multiple ways. Restraining the hypoxia/HIF axis offers a potential therapeutic approach that might synergize with immunotherapy in HCC (*48*). Coincidentally, HCC progression and metastasis are closely related to altered mitochondrial metabolism, and mitochondrial oxidative phosphorylation defects and ROS production are attributed to mitochondrial dysfunction (*49*). A critical step involves assessing the expression levels of COX IV, which governs electron transfer efficiency and the stabilization of the proton gradient within the oxidative phosphorylation system. GPX4, which can reduce the level of ROS, is a promising target for cancer cells under therapy-resistant HCC conditions (*50*). The results of Western blot analysis show that, compared with the control group, US, NVs, and miltirone-NVs groups, the expression of HIF-1α and GPX4 is markedly suppressed in the MBZO-NVs and MBZO/M-NVs with US groups and accompanied by COX IV down-regulation in the miltirone-NVs and MBZO/M-NVs with US irradiation groups (Fig. 5H). The synergistic therapeutic effects of MBZO/M-NVs induce hypoxia release, lipid peroxidation, and mitochondrial dysfunction, targeting metabolism and reprogramming the immunosuppressive microenvironment, which directly or indirectly leads to a cytotoxic effect in HCC.

To intuitively observe the PCT properties and curative effect of MBZO/M-NVs, the cytotoxicity, intracellular ROS generation, hypoxia release potential, and induction of mitochondrial dysfunction by the treatments were further determined using fluorescence probes. The calcein acetoxymethyl ester (calcein-AM) and propidium iodide (PI) double staining assay reveals that the Ctrl, US, and NVs groups exhibit a minimal extent of PCD. In contrast, most of the cells died in the miltirone-NVs, MBZO-NVs, and MBZO/M-NVs with US irradiation groups (Fig. 5I). 2′,7′-Dichlorodihydrofluorescein diacetate (DCFH-DA), which can be oxidized to 2,7-dichlorofluorescein with green fluorescence, was used to detect substantial ROS generation via the curative effect of miltirone and PCT by MBZO with US irradiation. The miltirone-NVs, MBZO-NVs+US, and MBZO/M-NVs+US groups exhibit stronger green fluorescence, indicating that the generation of endogenous and exogenous ROS is attributed to the miltirone-stimulating and PCT effects, respectively (Fig. 5J). The oxygen generation ability of the MBZO nanocubes in Huh7 cells was also demonstrated by [Ru(dpp)_3_]^2+^Cl_2_, an indicator for probing intracellular oxygen generation, which can react with oxygen to cause a fluorescence burst, thus confirming the oxygen content. The red fluorescence in the presence of MBZO-NVs and MBZO/M-NVs with US groups is significantly reduced, which can be attributed to the enhanced oxygen generation for hypoxia release and reprogram the immunosuppressive condition (Fig. 5K). Given the critical role of mitochondria in regulating the bioprocess of HCC, mitochondrial membrane potential (ΔΨ_m_) was measured using JC-1, a fluorescent dye sensitive to mitochondrial membrane potential. Compared with the control and US groups, Huh7 cells treated with miltirone-NVs, MBZO-NVs with US irradiation, and MBZO/M-NVs with US irradiation exhibit evident green fluorescence, indicating severe membrane potential depolarization and mitochondrial destabilization (Fig. 5L). Excessive production of ROS can effectively disrupt mitochondrial function by exacerbating oxidative stress, thereby inducing PCD (*15*). Last, the annexin V–FITC and PI double staining assay kit was used to evaluate the ability of treatments to induce cell PCD, especially the development of apoptosis (Fig. 5M). Apoptosis quantification via annexin V–FITC/PI staining revealed MBZO/M-NVs+US induced (Q2+Q3)% up to (73.41 ± 8.69)%, surpassing other groups: US (9.47 ± 5.69)%, NVs (4.58 ± 3.90)%, miltirone-NVs (32.56 ± 3.82)%, and MBZO-NVs+US (38.66 ± 5.38)% (fig. S16). Collectively, multimodal analyses establish the piezobiomimetic delivery nanosystem as an effective in vitro tumoricidal agent through coordinated mechanisms of hypoxia release, damage to the oxidative phosphorylation system, and mitochondrial dysfunction, thereby reprogramming the immunosuppressive condition and inducing PCD.

### Immunogenic PANoptosis activation

**Fig. 6.**
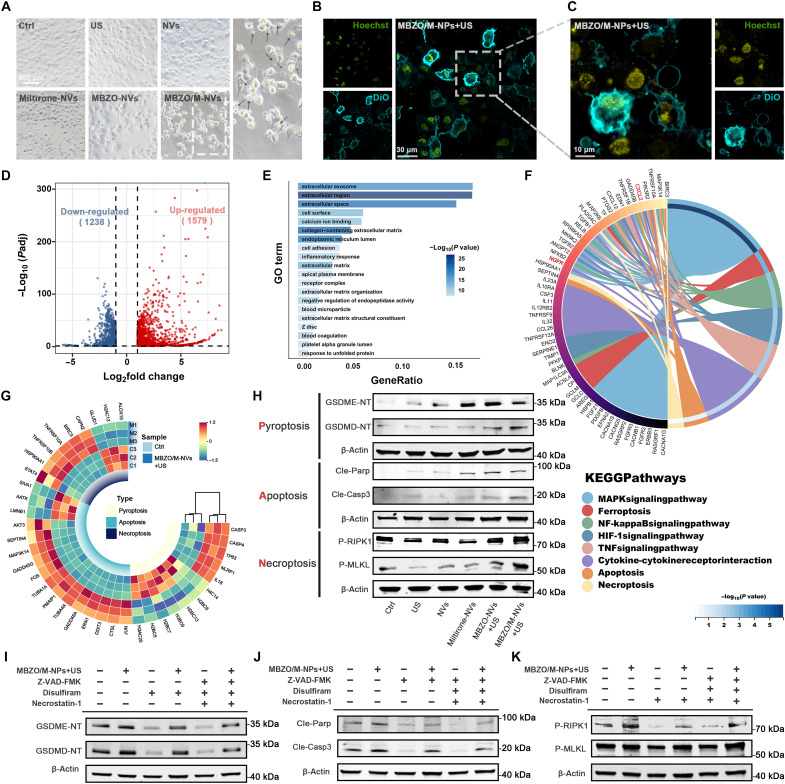
Molecular mechanism of MBZO/M-NVs–mediated PANoptosis effect. (**A**) Typical photographs of Huh7 cells after treatments. The black arrows indicate the damage to Huh7 cells pretreated. (**B**) Representative CLSM images of the morphology of Huh7 cells treated with MBZO/M-NVs+US and (**C**) enlarged insight of the bubbling from the DiO-labeled Huh7 cell membrane. (**D**) Volcano plot of DEGs identified in untreated and irradiated cells in the MBZO/M-NVs+US group. (**E**) GO term of DEGs and (**F**) KEGG pathway enrichment analysis for the identified top 300 DEGs between the two groups. (**G**) Heatmap of DEGs associated with PANoptosis. (**H**) Western blot analysis was used to assess the protein expression of representative pyroptosis-related, apoptosis-related, and necroptosis-related proteins in Huh7 cells following various treatments. Western blotting analyzed the related proteins of (**I**) pyroptosis, (**J**) apoptosis, and (**K**) necroptosis in Huh7 cells treated with MBZO/M-NVs+US and/or Z-VAD-FMK, disulfiram, and necrostatin-1.

Building on the demonstrated PCT efficiency in reprogramming the immunosuppressive condition and inducing PCD, it was necessary to characterize the cell death modalities and mechanisms systematically. Representative microscopy reveals distinct morphological alterations in Huh7 cells across treatment groups. Beyond partial apoptotic hallmarks of cellular shrinkage and cytoplasmic condensation, a major proportion of cells treated with MBZO/M-NVs+US exhibited a spectrum of atypical morphological alterations indicative of PCD pathways activation, including pronounced cellular edema, membrane blebbing, and structural disintegration (denoted by black arrowheads) (Fig. 6A). Subsequent CLSM analysis of 3′,3′-dioctadecyloxacarbocyanine perchlorate (DiO)–stained plasma membranes in MBZO/M-NVs with US irradiation–treated group confirms the pathological transformations, demonstrating characteristic membrane ballooning with compromised barrier function and progressive loss of architectural integrity (Fig. 6, B and C). The morphometric signatures, particularly the macrovesicular protrusions, align with pathognomonic features of PCD pathways, such as pyroptosis, necroptosis, and PANoptosis, suggesting the activation of inflammatory cell death mechanisms.

Transcriptomic profiling was performed using RNA sequencing to compare untreated cells with MBZO/M-NVs+US–treated cells, aiming to elucidate the molecular mechanisms underlying MBZO/M-NVs–induced cell death. The volcano plot shows differentially expressed genes (DEGs) were detected, in which 1579 genes were up-regulated whereas 1238 genes were down-regulated (Fig. 6D). Gene Ontology (GO) enrichment reveals pronounced enrichment of DEGs in biological processes and cellular component associated with inflammatory response and the extracellular matrix (ECM) (Fig. 6E). The ECM can inhibit T cell infiltration into the tumor core, impeding the infiltration of CD4^+^ and CD8^+^ T cells into the tumor core (*6*, *51*). Complementary gene set enrichment analysis (GSEA) corroborates these findings, showing effective activation of inflammatory response and adaptive immune response–related processes (fig. S17A). Next, DEGs associated with inflammatory response and adaptive immune response were picked up and analyzed in a protein-protein interaction (PPI) network. The topological analysis identifies chemokine ligand 2 (CXCL2) and Toll-like receptor 4 (TLR4), which are typical inflammation-related genes, as critical network hubs with extensive interaction degrees, highlighting the stimulation of immunogenic factors in HCC cells and potential influence on the chemotactic capacity of immune cells (fig. S17B). Studies have shown that the DAMP molecule, high-mobility group box 1 (HMGB1), is increased in peripheral blood samples and induces CXCL2 production in nonclassical monocytes via TLR4/MyD88. The release of granules by mast cells and the synthesis of CXCL1/CXCL2 are TLR4 dependent (*52*, *53*). Kyoto Encyclopedia of Genes and Genomes (KEGG) pathway analysis further demonstrates the enrichment of DEGs on PCD pathways, particularly pyroptosis and necroptosis, and other inflammatory-related signaling pathways, such as nuclear factor κB (NF-κB) signaling pathway and tumor necrosis factor (TNF) signaling pathway (Fig. 6F). Increases in NF-κB activation, pro-inflammatory cytokine production, and expression of NF-κB–dependent genes are induced by TLR4 stimulation (*54*). Key multifunctional regulators on KEGG pathways, such as NGFR (nerve growth factor receptor) and CXCL2, exhibit up-regulated expression after treatment, which was validated at the protein level through Western blotting (fig. S18, A and B). Clinical correlation analyses of RNA sequencing data from 364 patients with HCC demonstrate that elevated expression of NGFR [hazard ratio (HR) = 0.58; 95% confidence interval (CI): 0.41 to 0.82] and CXCL2 (HR = 0.67; 95% CI: 0.48 to 0.93) are associated with prolonged relapse-free survival. The bioinformatics analyses suggest that the piezobiomimetic delivery nanosystem triggered the activation of immunogenic PCD pathways and tumor-associated inflammatory responses, reprogrammed the immunosuppressive condition by restructuring the ECM, and modulated the expression of prognostic biomarkers to redefine the piezoimmunotherapy strategy (fig. S18C).

Moreover, DEGs associated with PCD pathways, such as apoptosis, pyroptosis, and necroptosis, were clustered in the heatmaps, demonstrating concurrent activation of PANoptosis (Fig. 6G). PANoptosis, a recently defined immunogenic death paradigm, involves PANoptosome complex formation that orchestrates caspase-mediated apoptosis, gasdermin-driven pyroptosis, and receptor-interacting protein kinase (RIPK)/mixed lineage kinase domain-like protein (MLKL)–dependent necroptosis through shared upstream sensors (e.g., ZBP1 and AIM2) (*21*). To deeply verify the possible cell death patterns (PANoptosis), Western blot analysis confirms proteolytic cleavage of pyroptotic effectors GSDME-N-terminal (GSDME-NT) and GSDMD-N-terminal (GSDMD-NT) in the MBZO/M-NVs+US group, indicating gasdermin-mediated membrane pore formation. Concurrently, apoptotic markers, cleaved caspase-3 and cleaved PARP-1, show effective up-regulation, confirming the activation of apoptosis. Necroptosis is an inflammatory form of PCD requiring RIPK1 and RIPK3 and MLKL. The kinase of RIPK3 phosphorylates MLKL, causing MLKL to form pore-like structures that allow the release of intracellular contents and lead to cell death (*55*). Intriguingly, the MBZO/M-NVs+US–treated cells exhibit significantly increased expressions of p-RIPK1 and p-MLKL (Fig. 6H). The results demonstrated that necroptosis also existed in combination with PCT by activating the RIPK1/MLKL pathway. Multiple cell death pathways, intertwining PANoptosis, were activated in the therapy. Pharmacological inhibition assays using Z-VAD-FMK (apoptosis), necrostatin-1 (necroptosis), and disulfiram (pyroptosis) failed to alter the expression of representative proteins (Fig. 6, I to K), confirming that PANoptosis induced by the treatment of MBZO/M-NVs+US is a molecularly integrated process rather than the additive activation of three pathways. The coordinated activation of pyroptotic (GSDME/D-NT), apoptotic (caspase-3/PARP-1 activation), and necroptotic (p-RIPK1/p-MLKL) effectors establishes MBZO/M-NVs+US as a potent inducer of PANoptosis. The piezobiomimetic delivery nanosystem synergistically induces cytotoxicity by hypoxia release, lipid peroxidation, and mitochondrial dysfunction while concurrently eliciting pro-inflammatory responses and an adaptive immune response via activation of PANoptosis against HCC.

### DC activation by PANoptosis and STING pathway–mediated DAMPs

**Fig. 7.**
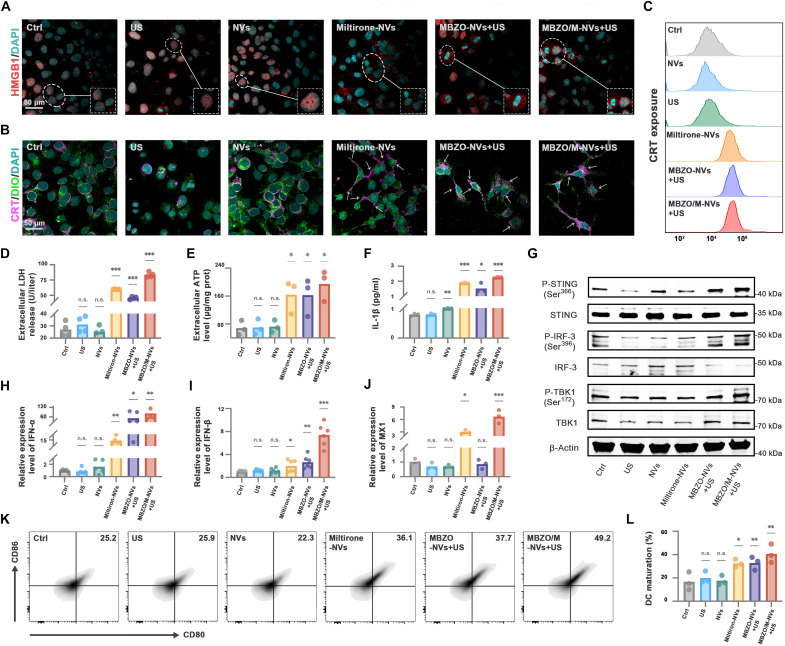
PANoptosis and STING signaling pathway–amplified ICD effects. (**A**) CLSM images showed HMGB1 migration from the cell nuclei and (**B**) CRT exposure on the cell surface after different treatments. (**C**) Flow cytometry analysis of CRT exposure on Huh7 cells with different treatments. (**D** and **E**) Measurement of extracellular release of (D) LDH and (E) ATP. (**F**) ELISA quantification of secreted pro-inflammatory cytokines IL-1β. (**G**) Western blot analysis of STING pathway–related proteins. (**H** to **J**) qPCR analysis of the relative mRNA level of (H) IFN-α, (I) IFN-β, and (J) MX1. (**K**) Flow cytometry assay and (**L**) quantitative analysis of in vitro DC (CD11c^+^ CD80^+^ CD86^+^) maturation induced by treatments in the Transwell system. Data are presented as means ± SD (*n* = 3 to 6). Statistical analysis was determined using one-way ANOVA followed by Tukey’s post hoc test (*P < 0.05; **P < 0.01; ***P < 0.001; n.s., not significant).

PANoptosis is characterized by its intrinsic ICD properties, which robustly activate immune responses through the coordinated release of hallmark DAMPs, including the exposure of calreticulin (CRT) and the release of HMGB1 (*56*). The release of HMGB1 from the nucleus (magnification frame) into the extracellular space in miltirone-NVs, MBZO-NVs+US, and MBZO/M-NVs+US groups is evident from the CLSM analysis (Fig. 7A), concomitant with the elevated levels of CRT exposure on the cell surface (white arrow) (Fig. 7B), relative to the control, US, and NVs groups. Detailed analysis reveals that, although miltirone-NVs and MBZO-NVs+US treatments exhibit similar outcomes in promoting the release of HMGB1 (magnification frame) and the exposure of CRT (white arrow) in Huh7 cells, the morphological manifestations differed substantially. In miltirone-NVs–treated Huh7 cells, extensive HMGB1 protein translocation is observed from the nucleus to the cytoplasm and subsequently into the extracellular space. The difference is that the treatment of MBZO-NVs+US induces nuclear chromatin condensation with prominent cytoplasmic HMGB1 accumulation, displaying classical apoptotic morphology (magnification frame), suggesting that the US-induced ROS generation by MBZO initiates apoptosis. Notably, MBZO/M-NVs+US–treated cells exhibit a hybrid phenotype that combines features from both treatment modalities of MBZO-NVs+US and miltirone-NVs. Parallel observations are made in immunofluorescence imaging of CRT exposure. Miltirone-NVs–treated cells maintained their cell morphology and had DiO-labeled plasma membranes that completely encapsulated the nuclei. However, the cells exhibited cellular shrinkage, accompanied by CRT transposition on the membrane surface and the outward protrusion of bubble-like structures simultaneously. Given the functions of miltirone as a pyroptosis inducer in HCC cells, the morphological alterations likely represent GSDME-NT/GSDMD-NT–mediated pore formation of the cell membrane, facilitating the release of cytoplasmic content through the pore at the late stage of PANoptosis. MBZO-NVs+US treatment induced CRT translocation and membrane disruption but with distinct pathological characteristics, including severe membrane damage and complete loss of cellular structures. The observed membrane damage correlates with elevated p-MLKL expression in the MBZO-NVs and MBZO/M-NVs groups, as determined by Western blot analysis, suggesting that stimulation of MBZO initiates necroptosis through MLKL phosphorylation–mediated membrane disruption. The coordinated mechanisms, including the proliferation of ROS-driven apoptosis, the release of HCC pyroptosis inducer miltirone-driven pyroptosis, and the accumulation of MBZO-driven necroptosis, explain the shared endpoint of DAMP release, despite their distinct morphological trajectories, with integrated manifestations observed in the MBZO/M-NVs+US treatment group. At the same time, the flow cytometry results further reveal predominant CRT-exposing cells in the MBZO/M-NVs+US group (Fig. 7C).

Notably, pyroptosis is an inflammatory PCD characterized by membrane perforation, cell swelling, and leakage of cellular contents, such as adenosine triphosphate (ATP), lactate dehydrogenase (LDH), interleukin-1β (IL-1β), and interleukin-18 (IL-18) (*31*, *56*). Thus, the released LDH, ATP, and pro-inflammatory cytokines related to pyroptosis, including IL-1β and IL-18, in the cellular supernatant were further detected. The released LDH and ATP levels in the MBZO/M-NVs+US group are much higher than those in other groups (Fig. 7, D and E). Meanwhile, the enzyme-linked immunosorbent assays (ELISAs) show that the release of extracellular inflammatory factors, including IL-1β and IL-18, is markedly increased in the MBZO/M-NVs+US group (Fig. 7F and fig. S19A). The results conclusively demonstrate that the piezobiomimetic delivery nanosystem treatment triggered the release of DAMPs in HCC cells via the induction of PANoptosis, promoting immunogenicity.

However, the heterogeneity of DCs in liver malignancies restricts antigen presentation, which has the ability to compromise the efficacy of PANoptosis-mediated immunotherapies. The Mn element has been demonstrated to potentiate DC maturation and innate immune activation through STING pathway stimulation (*31*, *57*). Therefore, the engineered piezobiomimetic delivery nanosystem not only induces PANoptosis but also enhances its immune-activating ability by MBZO nanocubes, which activate the STING pathway. Western blot analysis reveals significant up-regulation of phosphorylated STING (p-STING) in MBZO-treated groups compared to control, US, NVs, and miltirone-NVs groups (Fig. 7G). The phosphorylation cascade triggered sequential activation of downstream effectors, including TANK-binding kinase 1 (TBK1) phosphorylation (p-TBK1), which facilitated interferon regulatory factor 3 (IRF3) phosphorylation (p-IRF3). The processes establish mechanistic validation of STING pathway engagement and demonstrate successful activation of the STING pathway by MBZO treatment. In addition, the downsteam products of the STING signaling pathway, including interferon-α (IFN-α) and interferon-β (IFN-β) in the cells treated with MBZO, are increased (Fig. 7, H and I). The quantitative reverse transcription polymerase chain reaction (qRT-PCR) analysis of myxovirus resistance protein 1 (MX1), a canonical ISG and the STING signaling amplifier, shows pronounced up-regulation in the MBZO/M-NVs+US group (Fig. 7J). The findings suggest a self-reinforcing immunomodulatory loop; MBZO-activated STING signaling enhances MX1 expression, which, in turn, amplifies IFN-dependent immune activation through positive feedback regulation. Furthermore, exposure to conditioned media from MBZO/M-NVs+US–treated cells significantly enhanced tumor necrosis factor–α (TNF-α) secretion in mature DCs (fig. S19B), correlating with phenotypic maturation markers. The immunogenicity of dying cells undergoing the PANoptosis process and the secretion of ISGs were further assessed using an in vitro DC maturation assay. Bone marrow–derived DCs isolated from C57BL/6N mice were incubated for 24 hours with supernatants from Huh7 cells after treatments. Minimal DC maturation is observed after incubation with the supernatant of cells in the Ctrl, US, and NVs groups, likely due to insufficient immunogenic stimuli. Notably, the DC maturation frequency increases to 49.2% when DCs are treated with the supernatant of cells in the MBZO/M-NVs+US group (Fig. 7, K and L), demonstrating synergistic immunostimulation induced by DAMPs released during PANoptosis and activation of the STING signaling pathway. Collectively, the piezobiomimetic delivery nanosystem could serve as an excellent dual activator of PANoptosis and the STING signaling pathway. The parallel activation of the PANoptosis/STING pathway could efficiently amplify tumor immunogenicity through the release of abundant DAMPs, thereby triggering the EICD to foster the maturation of DCs and enhance antigen presentation, which, in turn, lays the groundwork for inducing ICD.

### CT and MRI properties

**Fig. 8.**
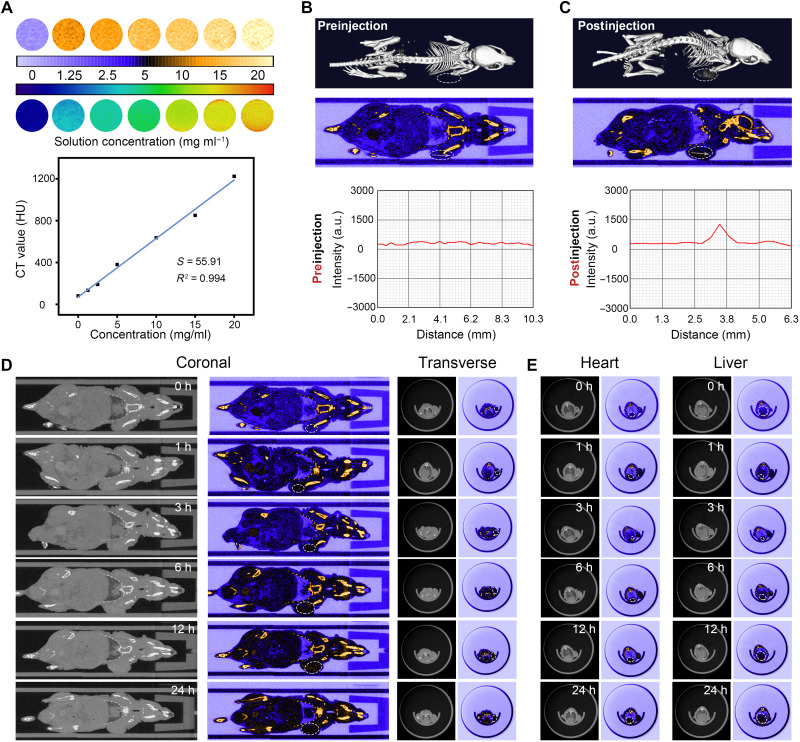
Evaluation of in vitro and in vivo imaging properties. (**A**) In vitro CT images (top) and corresponding CT values (bottom) were proportional to MBZO/M-NVs concentrations. HU, Hounsfield units. (**B** and **C**) In vivo CT images (top) of tumor-bearing mice (B) before or (C) after intratumoral injection and line profiles (bottom) composed of CT values corresponding to fault profiles. (**D**) CT images after injection of MBZO/M-NVs solution at different time points in vivo, including coronal sections and transverse slices. h, hours. (**E**) Coronal sections and transverse slices of the heart (left) and liver (right) in CT images.

The piezobiomimetic delivery nanosystem is a potential imaging contrast agent to guide the diagnosis and management of HCC. Computed tomography (CT) imaging relies on the x-ray mass attenuation effect of high-atomic-number elements. The high atomic numbers of Ba and Zr endow the materials with excellent x-ray attenuation properties. The mass attenuation coefficients are higher than those of conventional iodine-based contrast agents in the energy range commonly used for CT (50 to 150 keV), thereby enhancing the contrast of CT imaging by increasing the difference in x-ray absorption between tissues and the lesion site. The CT image grayscale gradually became white, and the pseudocolor images also changed to bright yellow as the concentrations of MBZO/M-NVs increased (Fig. 8A). As in the calculation results, the CT values are positively correlated with the concentrations of 0, 1.25, 2.5, 5.00, 10.00, 15.00, and 20.00 mg ml^−1^, and the Hounsfield unit value exhibited a high slope, indicating the outstanding ability as a CT imaging contrast agent of the piezobiomimetic delivery nanosystem. The high atomic numbers of elements Ba and Zr in the BaZrO lattice significantly increase the linear attenuation coefficients of local tissues under x-ray irradiation through the photoelectric effect, with Compton scattering dominating the attenuation process. Next, Huh7-bearing mice were injected with MBZO/M-NVs (100 μl per mouse, 10 mg ml^−1^), and digital CT images were obtained. The results of in vivo CT imaging on the mouse models reveal that the MBZO/M-NVs are enriched in diseased tissues, such as tumors, following intravenous injection (Fig. 8, B and C). The CT signals at the tumor site are sharply enhanced after 3 hours of intratumoral injection of MBZO/M-NVs, and the CT values of the tumor area are elevated two to three times compared to the preinjection values. In addition, the line profiles of CT values corresponding to fault profiles are confirmed in the conclusion.

Subsequently, MBZO/M-NVs were injected into the tail vein of Huh7-bearing mice. The CT images were also taken at different time points after intravenous injection. In vivo CT and pseudocolor images show that the signal intensity of tumor site reaches the highest value at 6 hours and remains significant 24 hours after injection (Fig. 8D). A substantial increase in signal contrast is observed in the liver at 3 and 6 hours after injection because MBZO/M-NVs are in the reticuloendothelial system of the liver at this time (Fig. 8E). Coating of homologous targeted NVs effectively reduces reticuloendothelial system uptake and prolongs blood half-life. In addition, clear CT signals are observed in both the bladder and intestine, suggesting that MBZO/M-NVs were primarily excreted through the hepatobiliary and renal systems and are less likely to cause damage to liver and kidney functions, thereby avoiding toxic side effects.

Moreover, the doping of Mn ions introduces a paramagnetic center. The unpaired d-orbital electrons of Mn ions could shorten the longitudinal relaxation time through both outer-sphere and inner-sphere effects, thereby enhancing weighted magnetic resonance imaging (MRI) signals. T1-weighted MRI images taken at different injection time points also show a trend of accumulation from 0 to 12 hours and gradual metabolism after 24 hours (fig. S20). Although the Mn element, as an essential trace element, achieves low systemic toxicity, the unpaired electrons in the Mn ions could generate a local magnetic field gradient, enhancing the signal of MRI. Therefore, MBZO/M-NVs could be used as multifunctional nanoprobes, and through the design of doping engineering and surface engineering, the synergistic optimization of CT/MRI dual-modality imaging functions can be achieved. The high contrast, low toxicity, and targeting capabilities provide a nanoprobe platform for precision medicine, integrating diagnosis and treatment. As a result, the outstanding CT and MRI imaging capabilities of the piezobiomimetic delivery nanosystem could promise a transformative leap from “rough detection” to “precise dynamic regulation” in tumor diagnosis and therapy.

### Systemic antitumor response

**Fig. 9.**
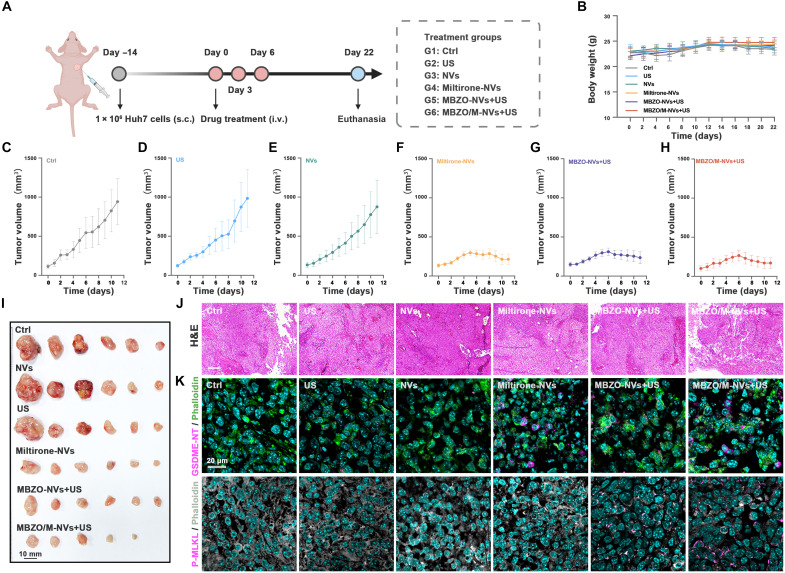
In vivo therapeutic performance. (**A**) Schematic illustration of setting a Huh7 xenotransplantation model. s.c., subcutaneous injection; i.v. intravenous injection. Created in BioRender. E, J. (2026) https://BioRender.com/wmo30tb. (**B**) Body weight of the Huh7 tumor-bearing mice after administration of treatments. (**C** to **H**) Individual tumor growth curves in groups. (**I**) Photograph of excised primary tumors at the end of treatments. (**J**) H&E staining (scale bar, 150 μm) of the tumor tissues receiving various treatments. (**K**) Confocal analysis of GSDME-NT or P-MLKL with phalloidin.

On the basis of the validation of dual activation of the PANoptosis and STING signaling pathways in vitro, and the evaluation of imaging properties in vivo, the antitumor efficacy of the piezobiomimetic delivery nanosystem was further studied on mouse models. A xenograft model was established by subcutaneously injecting Huh7 cells (8 × 10^6^) on the right dorsal side of each BALB/c-nu mouse. Huh7 tumor-bearing mice were randomly divided into the following groups (*n* = 6 per group), including Ctrl, US, NVs, miltirone-NVs, MBZO-NVs+US, and MBZO/M-NVs+US. Throughout the 22-day therapeutic regimen, MBZO/M-NVs were administered three times via intravenous injection on days 0, 3, and 6, whereas US irradiation (1.0 MHz, 1.0 W cm^−2^, and 30-s on and 30-s off for five on/off cycles) was applied after 6 hours of nanocubes injection during the initial 7-day period (Fig. 9A). Notably, no significant differences in body weight are observed among all groups during the treatment stage, indicating favorable biosafety profiles of the therapeutic modalities (Fig. 9B). Tumor growth analyses reveal that the tumors in the US and NVs groups grew rapidly without noticeable differences from those in the phosphate-buffered saline (PBS) group (Fig. 9, C to E). Moderate tumor suppression is achieved in the miltirone-NVs and MBZO-NVs groups (Fig. 9, F to H). Notably, the MBZO/M-NVs+US group exhibits the most potent tumor growth inhibition, as indicated by tumor volume measurements (Fig. 9I and fig. S21).

Furthermore, histopathological evaluation using hematoxylin and eosin (H&E) confirms that the treatment of MBZO/M-NVs+US apparently influenced the tumor malignancy grade, showing diminished nuclear atypia and decreased mitotic activity (Fig. 9J). The posttreatment status of heart, liver, spleen, lung, and kidney tissues in mice from each group was examined using H&E staining. The results showed that tissue integrity across all treatment groups, including US, NVs, miltirone-NVs, MBZO-NVs+US, and MBZO/M-NVs+US, remained comparable to that of the Ctrl group, with no significant damage observed, indicating the tumor specificity in the therapeutic effects and minimal side effects (fig. S22). In addition, GSDME-NT and P-MLKL, as the critical pore-forming proteins in the PANoptosis pathway, were the primary focus of the investigation, and phalloidin was used to visualize the cell outline as a tracker of the cytoskeleton. The results show that the US, NVs, and Ctrl groups exhibited similar profiles, with no significant cleavage of GSDME or phosphorylation of MLKL. The miltirone-NVs group demonstrated a moderate up-regulation of GSDME-NT, whereas the MBZO-NVs group showed increased P-MLKL expression. Notably, the MBZO/M-NVs group exhibits concurrent up-regulation of both GSDME-NT and P-MLKL (Fig. 9K). At the same time, immunofluorescence staining for Ki-67 (a cell proliferation-related protein) of tumors further confirms that the treatment of MBZO/M-NVs+US corroborated the antiproliferative effects. Meanwhile, the terminal deoxynucleotidyl transferase–mediated deoxyuridine triphosphate nick end labeling (TUNEL) assay performed on tumor sections presented extensive tumor apoptosis and necrosis in the group treated with the MBZO/M-NVs+US (fig. S23). The in vivo studies indicate that the piezobiomimetic delivery nanosystem could effectively disrupt the establishment of tumor structure through PANoptosis pathways, inhibit tumor proliferation, and enhance cytotoxicity for tumors.

### Convert cold tumors into hot ones

**Fig. 10.**
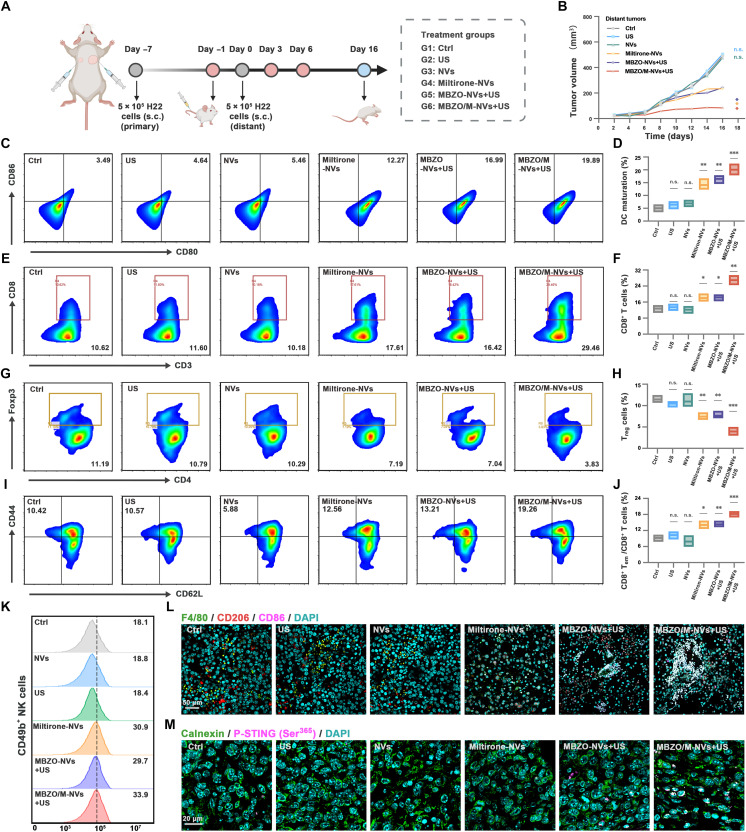
Immune evaluation triggered by MBZO/M-NVs. (**A**) Schematic representation of the therapeutic profile of H22 tumor-bearing mice. Created in BioRender. E, J. (2026) https://BioRender.com/wmo30tb. (**B**) Growth curves of distant tumors. Flow cytometry assays and quantitative analysis of (**C** and **D**) DC maturation in the draining lymph nodes, (**E** and **F**) CD8^+^ T cells (CD45^+^ CD3^+^ CD8a^+^), (**G** and **H**) T_reg_ cells (CD25^+^ CD4^+^ Foxp3^+^) in distant tumors, and (**I** and **J**) T_em_ cells (CD3^+^ CD8a^+^ CD44^+^ CD62L^−^) in the spleen. (**K**) Representative flow cytometric diagrams of infiltrated NK cells (CD3^−^ CD49b^+^) in distant tumors. (**L**) Merged images of immunofluorescence staining to explore the F4/80^+^ macrophage, including M1 macrophage (CD86^+^) or M2 macrophage (CD206^+^) infiltration in distant tumors. (**M**) Confocal analysis of P-STING with the endoplasmic reticulum marker protein calnexin. Data are presented as means ± SD (*n* = 3). Statistical analysis was determined using one-way ANOVA followed by Tukey’s post hoc test (*P < 0.05; **P < 0.01; ***P < 0.001; n.s., not significant).

The immunologically “cold” state tumor restricts cytolytic attack by tumor-infiltrating lymphocytes, resulting in a poor response to immunotherapy. To fully explore the EICD strategy of piezobiomimetic delivery nanosystem, antitumor immunity effect should be comprehensively evaluated, including T cell– and NK cell–mediated immune responses. On the basis of the superior in vivo antitumor efficacy and practical tumor-targeting ability of the piezobiomimetic delivery nanosystem, a bilateral H22 tumor-bearing model in BALB/c mice was used to evaluate the potential to enhance immune responses and improve the outcomes of systemic administration. The primary tumor was inoculated on the right dorsal side, followed by the inoculation of a second tumor on the contralateral dorsal side to simulate metastatic tumors. After 6 hours of each nanoparticle intravenous injection, US treatment was performed (Fig. 10A). Tumor growth analyses show that the treatment of MBZO/M-NVs+US remarkably resulted in higher tumor killing and inhibition in the distant tumors compared to other treatment groups in the therapeutic period (Fig. 10B). Subsequently, to evaluate the ICD-induced immune activation, the maturation of DCs was further investigated by collecting the tumor-draining lymph nodes of mice for flow cytometry analysis. Consistent with the in vitro trend observed in cultured DCs, no apparent changes in DC maturation are observed in the US group or NVs group, implying an unactivated ICD effect. By comparison, the proportions of matured DCs (CD11c^+^ CD80^+^ CD86^+^) in the miltirone-NVs, MBZO-NVs, and MBZO/M-NVs+US groups reach ~14.09, 16.47, and 20.07%, respectively (Fig. 10, C and D). The results reveal that the well-designed piezobiomimetic delivery nanosystem, functioning as an immunomodulator, has the potential to induce a robust immunostimulatory effect.

Mature DCs are recognized for their role in presenting tumor-associated antigens to lymphocytes, thus promoting the T cell infiltration into tumors. Correspondingly, the expression and activation levels of lymphocytes, including CD8^+^ T cells and regulatory T cells (T_reg_ cells) in distant tumors and effector memory T cells (T_em_ cells) in the spleen, were evaluated upon treatments to validate the stimulation of systemic adaptive immune responses. The treatments of miltirone-NVs and MBZO-NVs+US only display limited CD8^+^ T cells (CD45^+^ CD3^+^ CD8a^+^) infiltration in tumors, indicating limited immune activation in the immunosuppressive TME. The highest proportions of infiltrated CD8^+^ T cells in distant tumors are observed in the MBZO/M-NVs+US group, which is 2.1-fold higher than the CD8^+^ T cell infiltration of the Ctrl group, indicating the effective infiltration of cytotoxic T cells (CTLs) (Fig. 10, E and F). In addition, the expression of CD25, CD4, and Foxp3 proteins in tumor single cells was also examined. The results showed that treatment with MBZO/M-NVs+US induced a minimum number of T_reg_ cells (CD25^+^ CD4^+^ Foxp3^+^) compared to the other treatment groups (Fig. 10, G and H), confirming the minimal immunosuppression of T_reg_ cells on the antitumor efficacy. The results verify that the treatment of MBZO/M-NVs+US effectively converts cold tumors into hot ones, leading to tumor regression in HCC.

Moreover, the proportion of T_em_ cells (CD3^+^ CD8a^+^ CD44^+^ CD62L^−^) in the spleens of mice after different treatments was evaluated (Fig. 10I). Through counting, the MBZO/M-NVs+US group exhibits higher proportions of T_em_ cells than other groups (Fig. 10J). The results confirmed that the piezobiomimetic delivery nanosystem effectively promotes CTL proliferation, induces tumor-specific immunological memory responses, and reverses the immune “cold” TME. In addition to activated adaptive immunity, the intratumoral infiltration of NK cells (CD3^−^ CD49b^+^) in distant tumors increases to 33.9% in the MBZO/M-NVs+US group from 18.1% in the Ctrl group (Fig. 10K), suggesting the concurrently activated systemic innate immunity induced by localized DAMPs and promoting the priming, circulation, activity, trafficking, and fate of antitumor effector immune cells. In addition, both the mice in miltirone-NVs, MBZO-NVs, and MBZO/M-NVs groups show an effective influence on the serum levels of pro-inflammatory cytokines, including TNF-α (fig. S24A), IL-10 (fig. S24B), IFN-β (fig. S24C), and IFN-γ (fig. S24D), which are pivotal indicators of enhanced T cell immune responses, suggesting the robust activation of the immune system. Next, the enhanced tumor infiltration of CD4^+^ T cells (CD3^+^ CD4^+^) in distant tumors following treatment with MBZO/M-NVs+US was further verified by immunofluorescence staining (fig. S25). In addition to measuring IL-10, macrophage polarization was also assessed. The immunofluorescence staining images show that tumors treated with MBZO/M-NVs+US exhibit significantly increased infiltration of M1 macrophages (F4/80^+^ CD86^+^), which have the ability to kill tumor cells directly and promote the secretion of inflammatory factors and inhibit immunosuppressive cells. Notably, the level of M2 macrophage infiltration (F4/80^+^ CD206^+^) remained largely unaffected by the treatment, with substantial CD206^+^ M2 macrophage presence observed across all groups, suggesting that the therapeutic intervention may specifically drive changes in CD86^+^ M1 macrophage polarization without altering CD206^+^ M2 macrophage levels (Fig. 10L and fig. S26). Besides, through confocal analysis of P-STING (Ser^365^) and the endoplasmic reticulum marker calnexin, the result shows that the phosphorylation of the STING protein occurs in tumor tissues from both the MBZO/M-NVs and MBZO-NVs groups (Fig. 10M). The results, which were the same as the in vitro test, indicate that MBZO effectively promoted the activation of the STING pathway, with notably stronger P-STING signal intensity detected in the MBZO/M-NVs group. Collectively, the ingenious integration of NVs, MBZO, and miltirone within the immunomodulatory nanosystem of MBZO/M-NVs can systematically activate the STING pathway, driving DAMP release and antigen presentation, sensitize immunologically “cold” tumors to anticancer responses, bridge the robust systemic adaptive and the innate antitumor immunity, and trigger the EICD process, leading to practical tumor regression.

## DISCUSSION

This study aimed to construct a piezobiomimetic delivery nanosystem (MBZO/M-NVs) to address the challenge of converting immunologically “cold” HCC tumors into “hot” ones. By using doping defect–engineered manganese perovskite piezoelectric nanocubes as piezocatalysts for exogenous ROS generation, miltirone as a pyroptosis inducer and stimulus for endogenous ROS generation, as well as Huh7-derived NVs as a biomimetic delivery tool for biocompatibility, the system achieved parallel activation of PANoptosis and the STING pathway. Upon US stimuli, O_V_s-MBZO used the inherent piezoelectric properties and in situ TME conditions to trigger cellular oxidative stress. Concurrently, the loading of miltirone further amplified endogenous ROS generation and rapidly disrupted mitochondrial function, reshaping the immunologically “cold” TME properties of the HCC. The multistage ROS proliferation, MBZO accumulation, and pyroptosis inducer miltirone release jointly activated the PANoptosis process. Thus, the PANoptosis was synergistically activated to disrupt the cell barrier and release DAMPs, promoting immunogenicity. Simultaneously, MBZO enhanced the stimulation of the STING pathway, thereby evoking pro-inflammatory responses and eliciting ICD by triggering the EICD process. The parallel activation strategy reinvigorated CD8^+^ T cells with DCs, licensing them for CD8^+^ T cell priming and clonal expansion, and activated NK cells to kill tumor cells.

Previous studies have reported that PCT has emerged as a promising protocol for reprogramming the TME and enhancing the immune response to tumors and as a prospective noninvasive antitumor approach (*7*, *8*). Our findings align with recent advances in piezoelectric nanocatalysts for cancer therapy, where US-triggered ROS generation enhances oxidative stress and immunogenicity, and PCT-triggered hypoxia release remodels the TME. However, prior studies mainly focused on the activation of cell death pathways (*13*, *24*, *46*). Here, we demonstrate that parallel PANoptosis/STING activation induces a robust immune response. The STING pathway activation induced by Mn ions has been previously documented (*30*, *31*). However, the synergy effects of STING with the PANoptosis pathway to amplify DC maturation and T cell priming beyond additive effects were notable. In our study, the robust enhanced immune activation can be attributed to the concurrent release of DAMPs during PANoptosis, such as CRT exposure, HMGB1 secretion, and ATP release, which act as potent immunogenic signals. PANoptosis effectively augments tumor immunogenicity, whereas STING pathway activation further amplifies the antigen-presenting capacity of the immune system. This dual-action approach substantially improves the coordinated functionality of the antitumor immune response. A recent study aligns with our own regarding the activation of PANoptosis and the STING pathway. The study demonstrates that Zn^2+^ acts synergistically with ROS to trigger STING-AIM2-PANoptosis activation in a cascading manner (*58*). However, such practical therapeutic approaches still lack PCT strategy, spatiotemporal precision, and targeted delivery capabilities. In our analysis, the use of biomimetic NVs for homologous targeting further echoes strategies to improve nanoparticle accumulation in the TME, as seen in studies using biomembrane coatings for targeted delivery (*8*, *24*). The doping defect–engineered manganese perovskite nanocubes serve as a powerful piezocatalyst, generating ROS and oxygen under US irradiation. The incorporation of miltirone, a specific HCC pyroptosis inducer, further amplifies endogenous oxidative stress. This dual ROS and oxygen generation synergistically disrupts mitochondrial function, as evidenced by the loss of mitochondrial membrane potential and down-regulation of key metabolic proteins (HIF-1α, COX IV, and GPX4). This coordinated stress triggers a unique and effective form of inflammatory PCD, PANoptosis.

Our findings reveal that the piezobiomimetic delivery nanosystem induces a hybrid morphological phenotype in treated cells, like hallmark features of PANoptosis. This integrated cell death signature could not be replicated by any single form of PCD, as evidenced by the failure of pathway-specific inhibitors, including targeting apoptosis (Z-VAD-FMK), necroptosis (necrostatin-1), or pyroptosis (disulfiram), to alter the expression of key PANoptosis-related proteins. These findings suggest that the nanosystem triggers a cohesive PANoptotic process and irreversible reaction rather than a simple combination of independent death pathways. Furthermore, the immunological impact of this integrated death modality extended beyond local tumor control. In distant tumor sites, MBZO/M-NVs with US irradiation treatment effectively suppressed T_reg_ cells while enhancing the proliferation of CTLs and T_em_ cells, indicating the induction of a systemic antitumor immune response and memory. It is worth noting that, although the infiltration of CD206^+^ M2 macrophages remained almost unchanged across various treatment groups, levels of the immunosuppressive cytokine IL-10 were markedly reduced. Such a difference suggests a functional shift in macrophage behavior, possibly through the involvement of alternative M2 subtypes or a transition in polarization state, highlighting an unexpected immunomodulatory dimension of the piezobiomimetic delivery nanosystem.

The study supports the “EICD” model, wherein EICD, which encompasses the priming, circulation, activity, trafficking, and fate of antitumor effector immune cells, is a crucial step in transforming nonresponsive cold tumors into responsive hot ones (*6*). The nanosystem’s success aligns with the concept that coordinated innate and adaptive immune activation can overcome immunosuppressive barriers in HCC. Moreover, the piezobiomimetic delivery nanosystem demonstrates considerable potential as a theranostic platform. The high atomic numbers of Ba and Zr confer excellent x-ray attenuation properties, enabling clear CT imaging of tumor accumulation. The paramagnetic nature of the Mn element also offers potential for MRI. The dual-modal imaging capability allows for noninvasive monitoring of nanoparticle biodistribution and tumor targeting, which is crucial for guiding future therapeutic applications.

Several limitations of this study should be acknowledged. First, subcutaneous tumor models may not fully recapitulate the complexity of human HCC stroma and immune system. Then, long-term biosafety and nanoparticle accumulation require further toxicological profiling. Besides, the precise molecular mechanisms linking the initiation of the PANoptosome complex and the deployment of effector immune cells need further elucidation. Last, the scalability and reproducibility of the manufacturing process for the multicomponent nanosystems need to be addressed before clinical translation can be considered.

Together, the piezobiomimetic delivery nanosystem, which was designed to mitigate the immunosuppressive condition and treat cold tumors by parallel PANoptosis/STING pathway activation in HCC, successfully triggers the PCT and EICD processes, leading to valid tumor suppression and potent, systemic antitumor immunity. The integration of therapeutic and imaging functionalities further enhances its translational potential. Our study not only presents a promising nanoplatform for piezoimmunotherapy in HCC but also sheds light on the generalizable strategy for converting cold tumors into hot ones across cancers.

## MATERIALS AND METHODS

### Materials

Barium nitrate [Ba(NO_3_)_2_, ≥99.5% metal basis], zirconium tetrachloride (ZrCl_4_, ≥99.9% metal basis), manganese(II) nitrate tetrahydrate (MnN_2_O_6_·4H_2_O, ≥98% metal basis), TMB, and DTNB were purchased from Aladdin Scientific Co. Ltd. (Shanghai, China). Miltirone, FITC, DiI, DiO, and DCFH-DA were purchased from MedChemExpress Co. Ltd. (USA). Hoechst 33342, 4′,6-diamidino-2-phenylindole (DAPI), Mitochondrial membrane potential assay kit with JC-1, and TUNEL Apoptosis Assay Kit were obtained from Beyotime Biotechnology Co. Ltd. (Shanghai, China). CCK-8 reagent was obtained from Dojindo Laboratories (Japan). The calcein-AM/PI double staining kit was purchased from Beijing Solarbio Science & Technology Co. Ltd. (Beijing, China). [Ru(dpp)_3_]^2+^Cl_2_ was purchased from Bestbio (Nanjing, China). Annexin V–FITC Apoptosis Detection Kit and Phalloidin FITC were purchased from Yeason (Shanghai, China). ATP content detection kit was purchased from Service Technology Co. Ltd. (Wuhan, China). Bicinchoninic acid (BCA) Protein Assay Kit and TRIzol reagent, and SYBR green Premix EX Taq II were obtained from Thermo Fisher Scientific (USA). Human-Reactive STING Pathway Antibody Sampler Kit and Anti-P-STING (Ser365) antibody were obtained from Cell Signaling Technology (USA). Anti-GSDME antibody-N-terminal, Anti-cleaved N-terminal GSDMD antibody, and Anti-CXCL2 antibody were purchased from Abcam (USA). Anti-MLKL (Phospho Ser345) antibody and Anti-RIP (Phospho Ser166) antibody were obtained from Immunoway Biotechnology Co. Ltd. (USA). Anti-β-actin antibody, Anti-GPX4 antibody, Anti-HMGB1 antibody, Anti-Calreticulin, anti-Ki67 antibody, and anti-Calnexin antibody were obtained from Proteintech (Wuhan, China). Anti-HIF-1α antibody, Anti-COX IV antibody, Anti-Cleaved-PARP antibody, and Anti-Cleaved Caspase-3 antibody were obtained from Wanleibio Co. Ltd. (Shenyang, China). IRDye700 and IRDye800 were purchased from Licorbio Technology Co. Ltd. (USA). HiScript III RT SuperMix was obtained from Vazyme (Nanjing, China). Transwell system was obtained from Corning (USA). ELISA kits were obtained from Thermo Fisher Scientific (USA) and Multi Sciences Biotech (Hangzhou, China). Allophycocyanin (APC) anti-mouse CD11c antibody, FITC-CD80 anti-mouse antibody, phycoerythrin (PE) anti-mouse CD86, PerCP anti-mouse CD3, PE anti-mouse CD45, APC anti-mouse CD8a, FITC anti-mouse CD4, PE anti-mouse CD25, APC anti-mouse Foxp3, PE anti-mouse CD44, FITC anti-mouse CD62L, PE antimouse CD3, FITC anti-mouse CD49b, FITC anti-mouse F4/80, and APC anti-mouse CD206 were purchased from BioLegend (USA).

### Cell lines

Huh7 cells and H22 cells were procured from the Wuhan Pricella Biotechnology Co. Ltd. (CL-0120 and CL-0341, Wuhan, China) and cultured in Dulbecco’s modified Eagle’s medium (DMEM) and RPMI 1640, respectively (Gibco, USA). The culture media were supplemented with 10% fetal bovine serum (FBS) and 1% penicillin/streptomycin. The cells were maintained in the incubator at 37°C with a CO_2_ concentration of 5%.

### Animals

Male BALB/c nude mice (5 to 6 weeks, 20 to 24 g), male BALB/c mice (5 to 6 weeks, 18 to 22 g), and C57BL/6N mice were purchased from Beijing Vital River Laboratory Animal Technology Co. Ltd. (Beijing, China). All animal experiments were performed under the guidelines and approved by the ethics committee of Harbin Medical University (no. HMUIRB2025012PRE).

### Synthesis and characterization of piezonanocubes

A homogeneous mixture of Ba(NO_3_)_2_, ZrCl_4_, and MnN_2_O_6_·4H_2_O was dissolved in deionized water under continuous magnetic stirring. Subsequently, KOH solution was added dropwise to the mixture, followed by 30 min of constant agitation. The resulting suspension was transferred into a Teflon-lined hydrothermal autoclave and subjected to hydrothermal treatment at 160°C for 24 hours. After cooling to room temperature, the product was washed with deionized water and absolute ethanol through three cycles of dispersion and centrifugation. Last, the purified white precipitate was dried in an oven at 70°C for 12 hours to obtain the MBZO powder. The zeta potential of the nanocubes was determined using the dynamic light scattering (DLS) technique, whereas TEM was used to examine their morphology. XRD patterns were procured using an x-ray diffractometer across the scanning range of 10° to 90°. XPS and ICP-MS were used to determine the specific contents of MBZO.

### Preparation of MBZO/M-NVs

Huh7 cells were cultured with culture medium (non-FBS) for an additional 48 hours. Then, trypsin was used to harvest cells. The cell suspension was centrifuged, and the cell pellets were suspended in hypotonic buffer containing 225 mM d-mannitol, 30 mM tris-HCl (pH 7.5), 0.2 mM EGTA, 75 mM sucrose, and 1 mM PMSF (phenylmethylsulfonyl fluoride). Next, the Dounce homogenizer was used to disrupt cells for 30 to 50 passes under low-temperature conditions. Afterward, the suspension was centrifuged to discard cellular detritus. Last, the medium was filtered using a 0.22-μm pore filter. The NV pellets were resuspended with PBS and stored at −80°C. The solution was mixed with the mixture of miltirone and MBZO nanocubes and extruded through 400- and 220-nm polycarbonate membranes to form MBZO/M-NVs.

To explore the optimal ratio of NVs, miltirone, and MBZO nanocubes, we incubated them at different weight ratios (w/w) from 1:0.1:5 to 5:0.3:1 at 4°C for extrusion. Subsequently, the unloaded NVs and uncoated miltirone were removed via centrifugation for 30 min, and the collected MBZO/M-NVs were resuspended in PBS. To assess the optimized weight ratio, the surface membrane protein content of MBZO/M-NVs was determined by using the BCA kit. The DLS technique was used to determine the zeta potential of nanoparticles. Western blot detected protein expression profiles of Huh7-NVs and MBZO/M-NVs.

### In vivo antitumor efficacy

Thirty-six BALB/c nude mice were randomly divided into six groups (*n* = 6 per group) as follows: PBS, US, NVs, miltirone-NVs, MBZO-NVs+US, and MBZO/M-NVs+US. Mice were injected with 1 × 10^6^ Huh7 cells. When tumors grew to 60 mm^3^, they were intravenously administered with different drug formulations (5 mg kg^−1^). After 6 hours of intravenous injection with nanoparticles, US treatment was performed (1.0 MHz, 1.0 W cm^−2^, 30-s on and 30-s off for five on/off cycles). Afterward, the body weight and tumor volume were recorded every 2 days for 22 days. Tumor volume was calculated using the following formula: *V* = 0.5 × length × width^2^. Major organs and tumors were collected. Part of the tumors and organs were fixed in 4% paraformaldehyde (PFA), embedded in paraffin, and then used for H&E staining and immunofluorescence (TUNEL, Ki-67, GSDME-NT, and P-MLKL) before being imaged with a microscope.

### In vivo antitumor immune response

BALB/c mice bearing H22 bilateral tumors were randomly divided into six groups (*n* = 6 per group) as follows: PBS, US, NVs, miltirone-NVs, MBZO-NVs+US, and MBZO/M-NVs+US. H22-bearing mice were treated as described above. On day 16, the mice were euthanized, and the fresh lymph nodes, spleens, and tumors from each group were used to assess and prepare single-cell suspensions. Then, the cells were stained with flow cytometry antibodies (for T cells, PerCP anti-mouse CD3, PE anti-mouse CD45, APC anti-mouse CD8a; for T_reg_ cells, PE anti-mouse CD25, FITC anti-mouse CD4, and APC anti-mouse Foxp3; for T_em_ cells, PerCP anti-mouse CD3, APC anti-mouse CD8a, PE anti-mouse CD44, and FITC anti-mouse CD62L; and for NK cells, PE anti-mouse CD3 and FITC anti-mouse CD49b) following the instructions of the manufacturer and analyzed with the NovaExpress or FlowJo software.

### Statistical analysis

Data were presented as means ± SD (*n* = 3 to 6). Statistical analysis was conducted using a one-way analysis of variance (ANOVA) followed by Tukey’s post hoc test and multiple unpaired *t* tests for multiple comparisons. Statistical significance was determined at the following levels: n.s.: not significant, *P < 0.05, **P < 0.01, and ***P < 0.001.
