# Supplementary material for: Piezobiomimetic delivery nanosystem converts cold tumors to hot by parallel PANoptosis/STING activation in hepatocellular carcinoma
Source: Sci Adv. 2026 Jun 17;12(25):eaea6844. doi: 10.1126/sciadv.aea6844 (PMC13274619; doi:10.1126/sciadv.aea6844)
Supplement: Supplementary file 1 — Figs. S1 to S26 Tables S1 and S2 Supplementary Materials and Methods [file sciadv.aea6844_sm.pdf]

Science Advances
AAAS

Supplementary Materials for

Piezobiomimetic delivery nanosystem converts cold tumors to hot by parallel PANoptosis/STING activation in hepatocellular carcinoma

Jiaoting E *et al.*

Corresponding author: He Ding, dinghe@hrbeu.edu.cn; Jiuxin Zhu, zhujiuxin@hrbmu.edu.cn; Piaoping Yang, yangpiaoping@hrbeu.edu.cn; Rui Xie, rxie@hrbmu.edu.cn

Sci. Adv. **12**, eaea6844 (2026)
DOI: 10.1126/sciadv.aea6844

This PDF file includes:

Figs. S1 to S26
Tables S1 and S2
Supplementary Materials and Methods

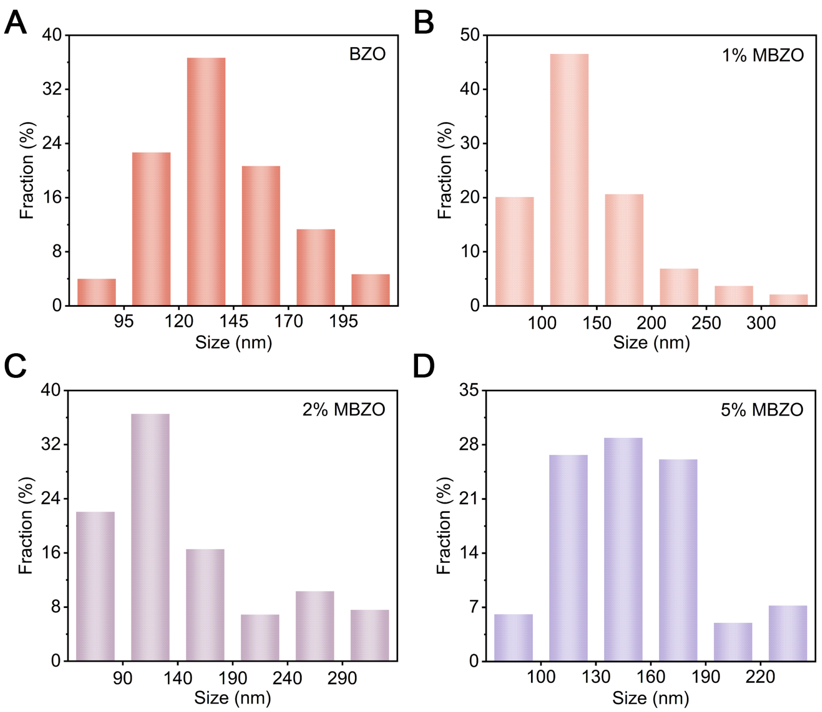

Fig. S1. (A-D) The average diameter of the **(A)** BZO, **(B)** 1% MBZO, **(C)** 2% MBZO, and **(D)** 5% MBZO nanocubes.

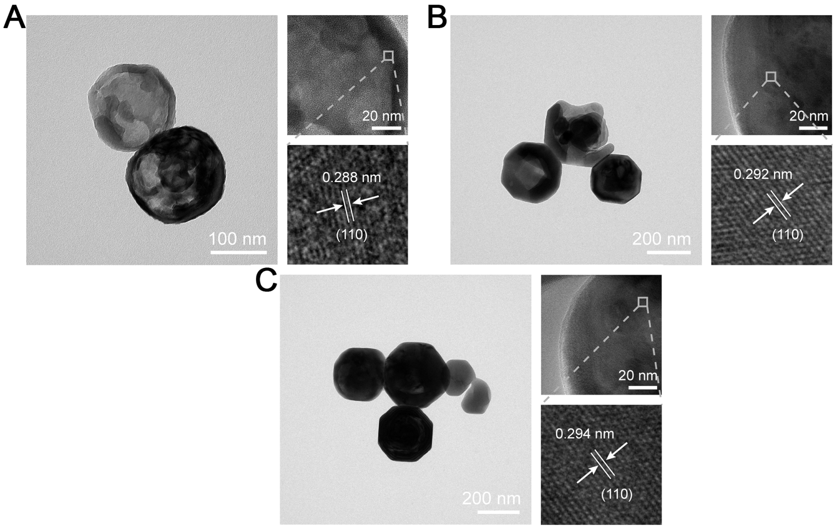

Fig. S2. (A-C) The lattice spacing of the same (110) plane for **(A)** BZO, **(B)** 1% MBZO, and **(C)** 2% MBZO nanocubes.

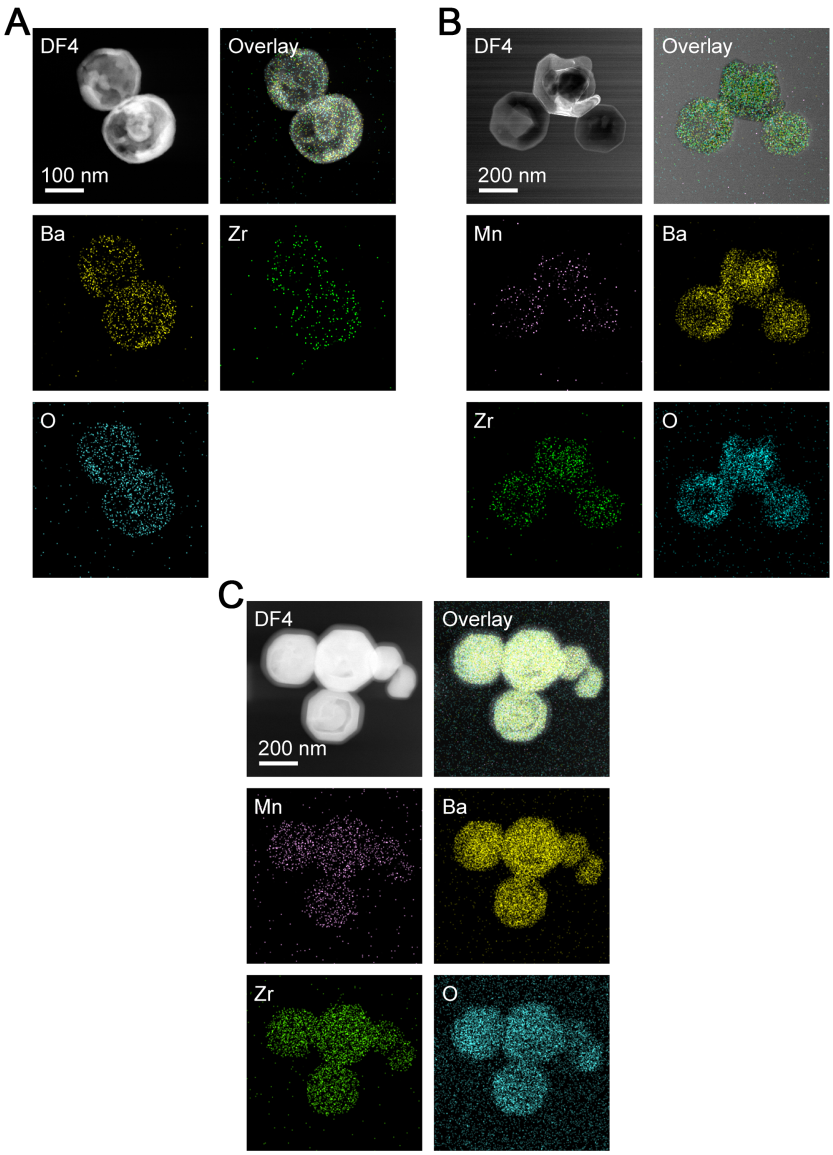

Fig. S3. (A-C) The morphology images and element distribution of **(A)** BZO, **(B)** 1% MBZO, and **(C)** 2% MBZO nanocubes.

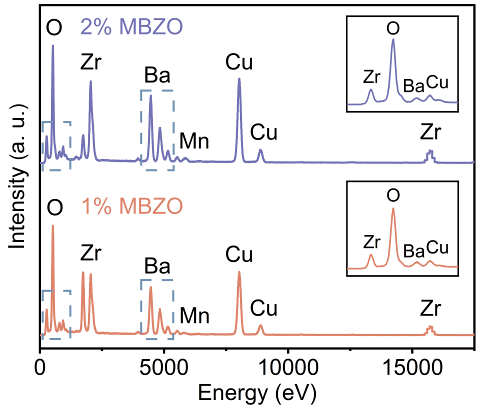

Fig. S4. The energy dispersive spectroscopy spectrum of 1% MBZO and 2% MBZO nanocubes.

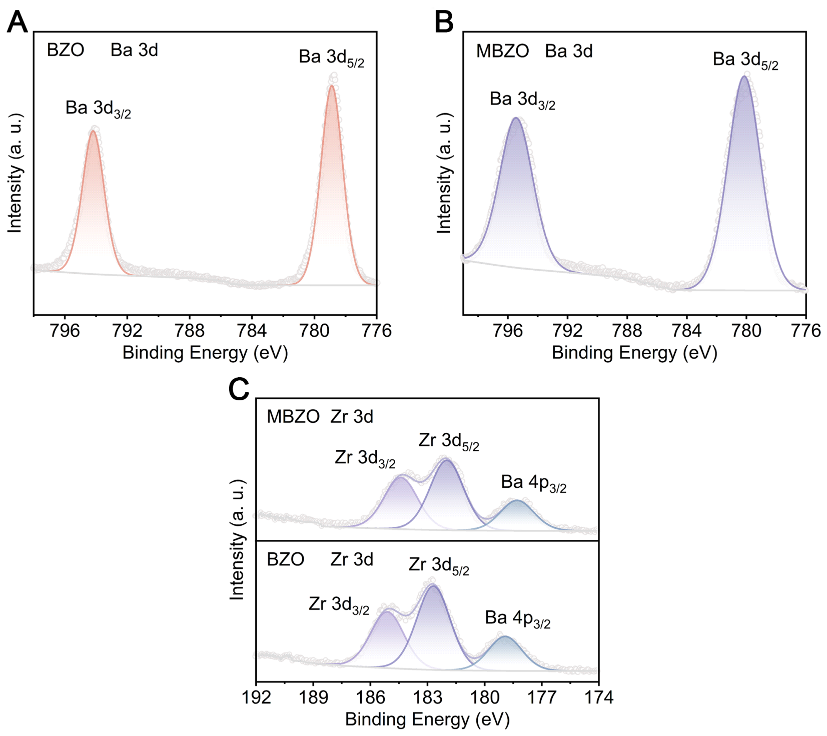

Fig. S5. (A, B) The high-resolution XPS spectra of Ba 3d in **(A)** BZO and **(B)** MBZO nanodubes. **(C)** The high-resolution XPS spectra of Zr 3d in BZO and MBZO nanodubes.

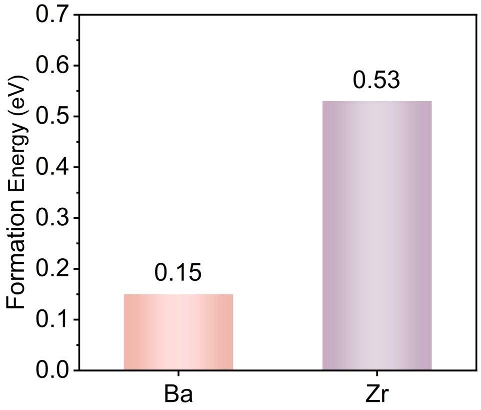

Fig. S6. The formation energy calculation of Mn doping sites in MBZO nanocubes.

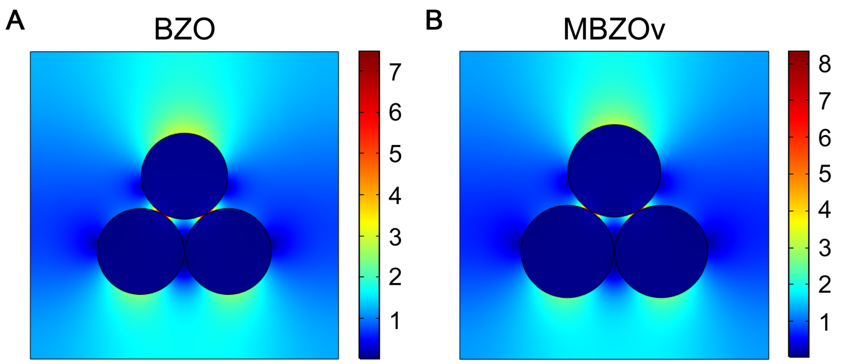

Fig. S7. (a, b) COMSOL simulation of **(a)** BZO and **(b)** MBZOv.

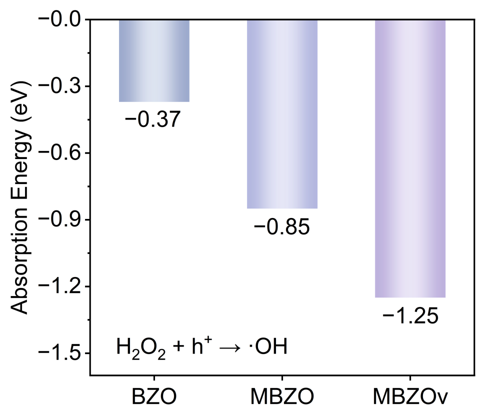

Fig. S8. The adsorption energy of H_2O_2 at the O_{VS} sites on the surface.

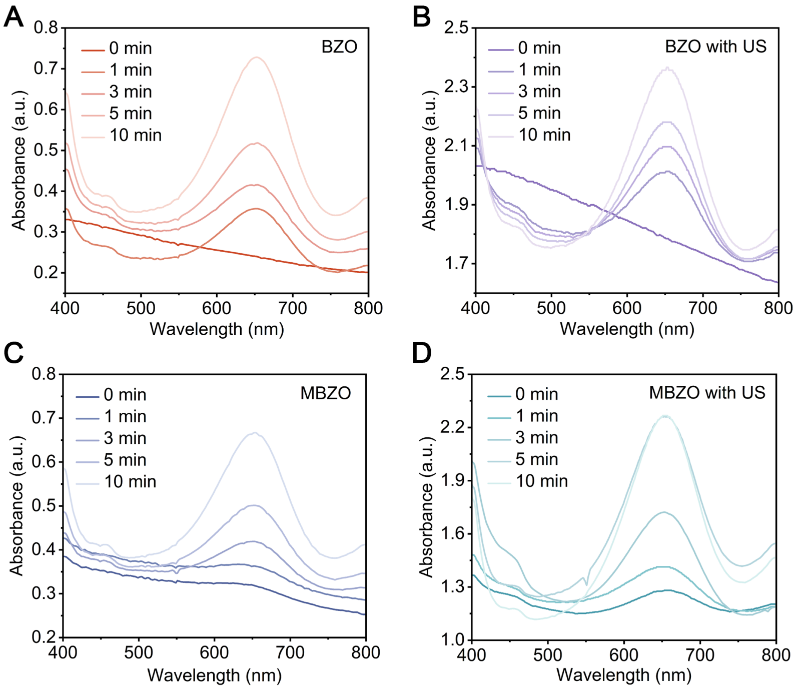

Fig. S9. (A-D) The time-dependent properties to generate ·OH of **(A)**BZO, **(B)** BZO with US, **(C)** MBZO, and **(D)** MBZO with US were verified by the color reaction of TMB at 652 nm.

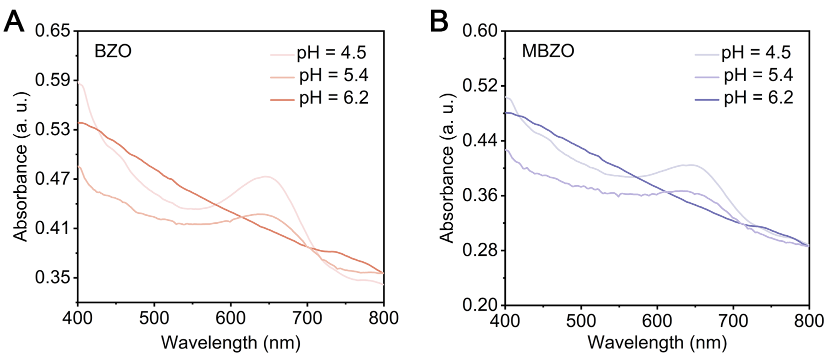

Fig. S10. (A, B) The properties to generate ·OH of **(A)**BZO with US and **(B)** MBZO with US were verified by the color reaction of TMB at different pH conditions.

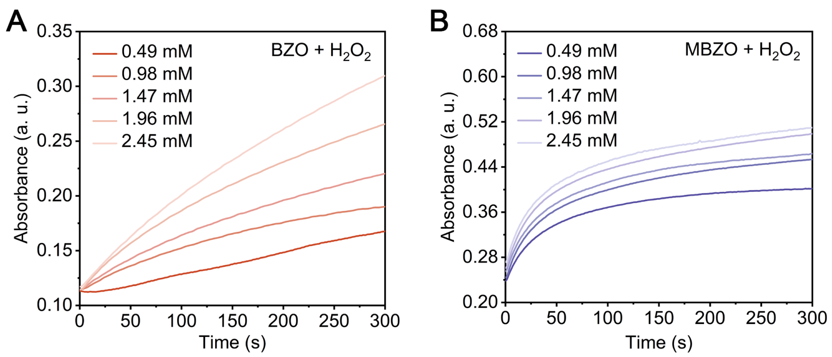

Fig. S11. (A, B) The initial reaction rate of steady-state kinetics analysis was determined with different concentrations of H_2O_2 in **(A)** BZO or **(B)** MBZO nanocubes.

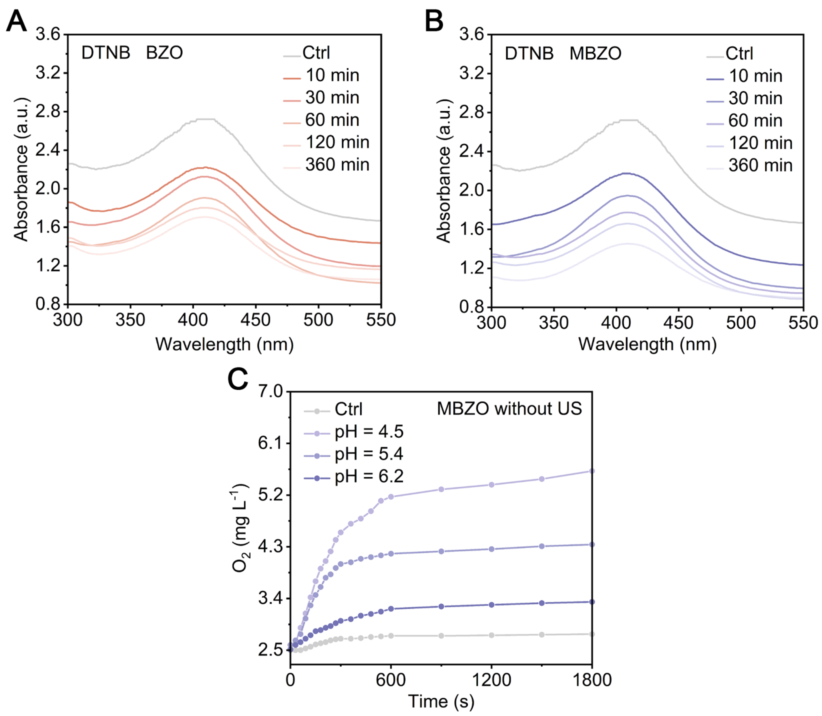

Fig. S12. (A, B) UV–vis absorption spectra of DTNB in **(A)** BZO or **(B)** MBZO nanocubes solution with US for different reaction times. **(C)** O_2 generation profiles of MBZO without US for different pH conditions.

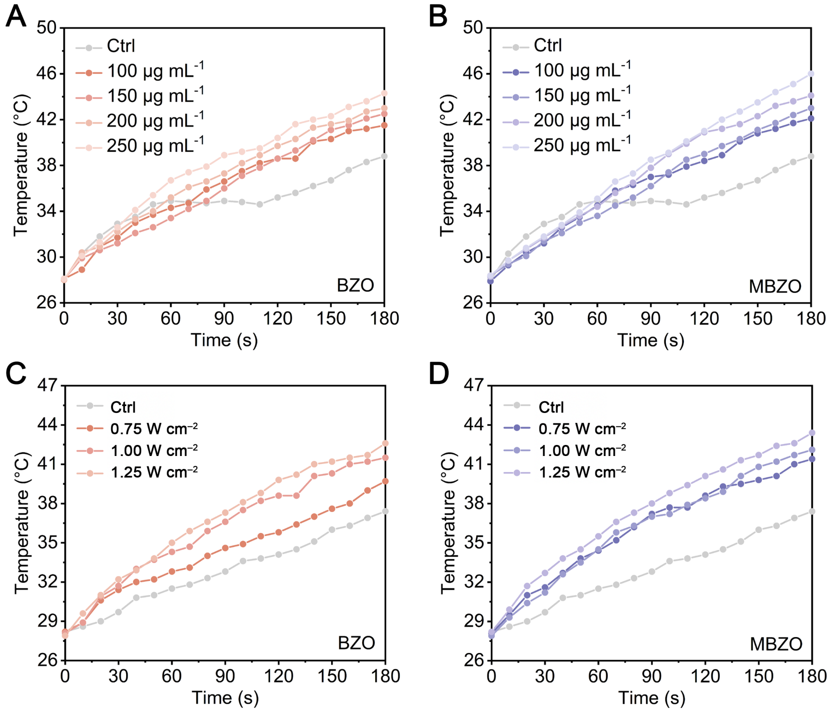

Fig. S13. (A, B) The heating curves of different concentrations of **(A)** BZO or **(B)** MBZO nanocubes with US irradiation. **(C, D)** The heating curves of varying power densities of **(C)** BZO and **(D)** MBZO nanocubes with US irradiation.

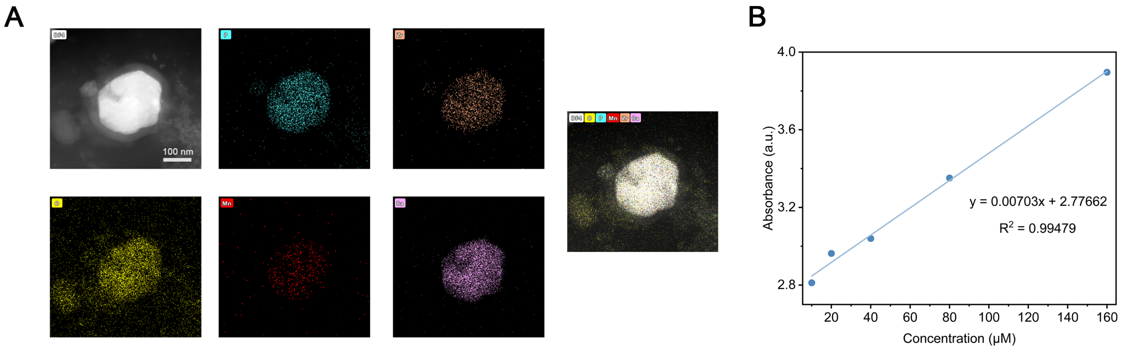

Fig. S14. (A) The morphology image and elemental mappings of MBZO/M-NVs. **(B)** The linear equation of concentration and absorbance of Miltirone.

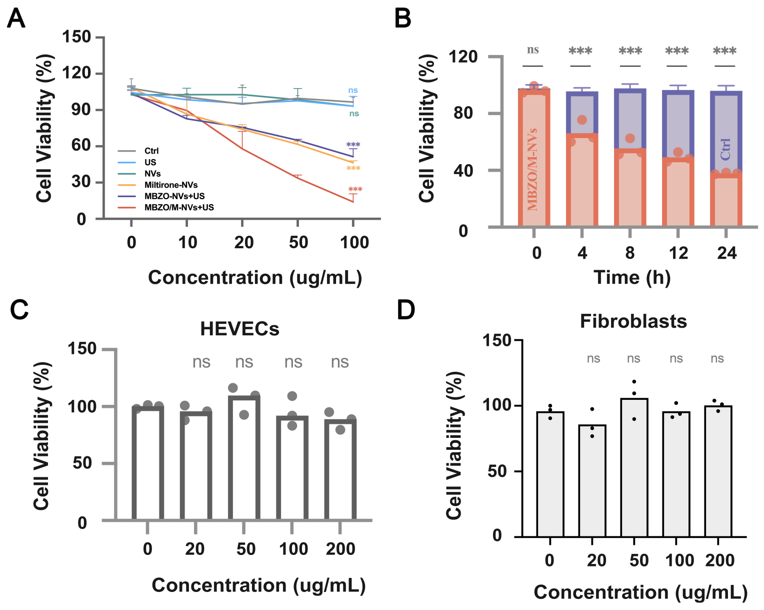

Fig. S15. (A, B) Cytotoxicity assays of Huh-7 cells treated with **(A)** various concentrations of nanosystem, and **(B)** different time lines of treatment. **(C, D)** Cytotoxicity assays of **(C)** human-derived HUVEC cells and **(D)** mouse-derived myocardial fibroblast cells treated with various concentrations MBZO/M-NVs with US. Data are presented as mean ± standard deviation (SD) (n = 3). Statistical analysis was determined using one-way ANOVA followed by Tukey's HSD and multiple unpaired t-test ($*P < 0.05$; $**P < 0.01$; $***P < 0.001$; n.s., no significance).

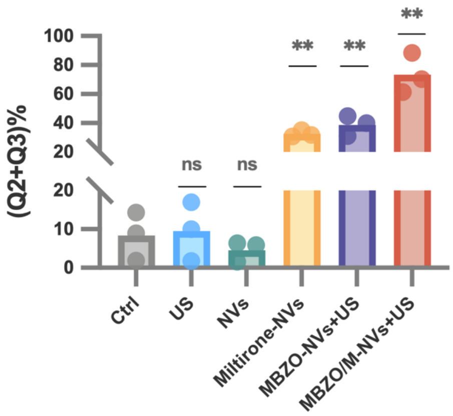

Fig. S16. Quantification analysis of apoptosis flow cytometry testing the Huh-7 cells subjected to different treatments. Data are presented as mean ± SD (n = 3). Statistical analysis was determined using one-way ANOVA followed by Tukey's HSD (*P < 0.05; **P < 0.01; ***P < 0.001; n.s., no significance).

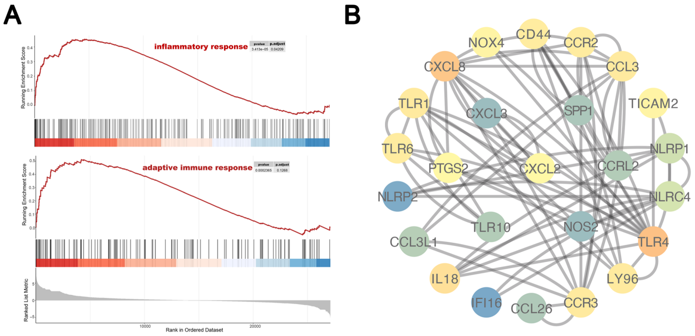

Fig. S17. (A) GSEA data sets of inflammatory response and adaptive immune response in the two groups. **(B)** PPI results of the proteins corresponding to the DEGs on the aforementioned GSEA data sets.

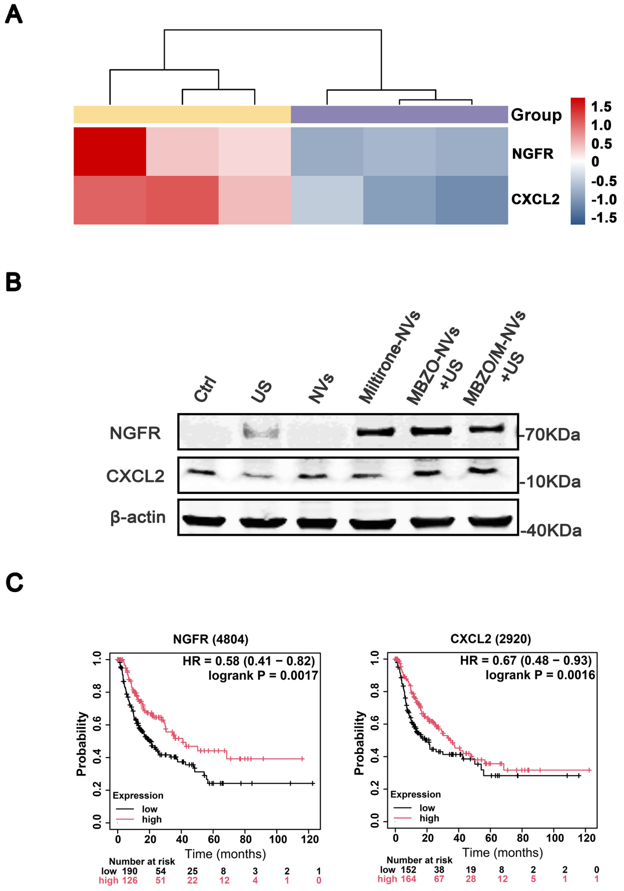

Fig. S18. (A) Heat map and **(B)** Western blot analyses of two representative genes on critical KEGG pathways. **(C)** The Kaplan-Meier plot of the correlation between the expression of NGFR(left) or CXCL2 (right) and the release-free survival in 364 patients from liver cancer RNA-seq datasets on the Kaplan-Meier plotter database.

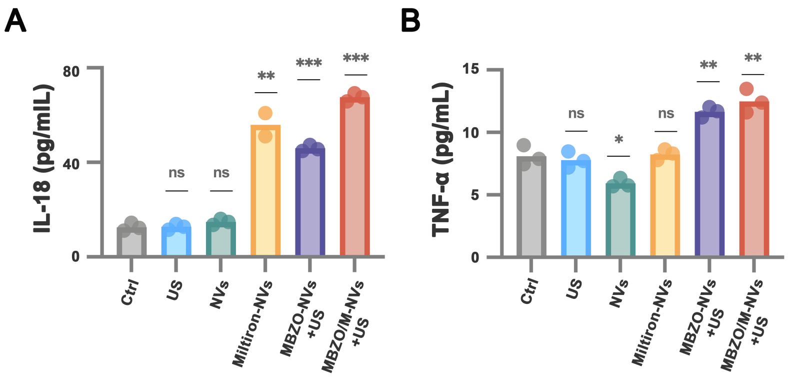

Fig. S19. (A, B) ELISA quantification of secreted pro-inflammatory cytokine **(A)** IL-18 and **(B)** TNF-α. Data are presented as mean ± SD (n = 3). Statistical analysis was determined using one-way ANOVA followed by Tukey's HSD (*$P < 0.05$; **$P < 0.01$; ***$P < 0.001$; n.s., not significance).

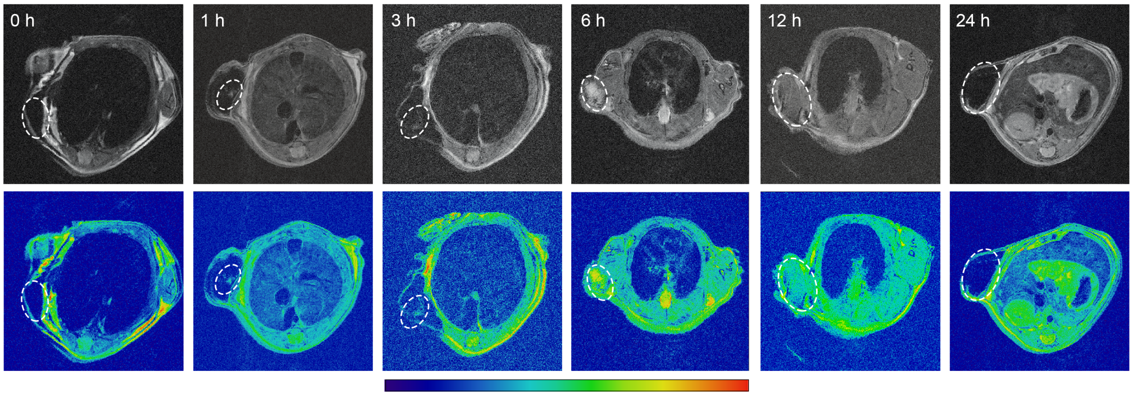

Fig. S20. In vivo MRI transverse slice images after injection of MBZO/M-NVs solution at different representative time points.

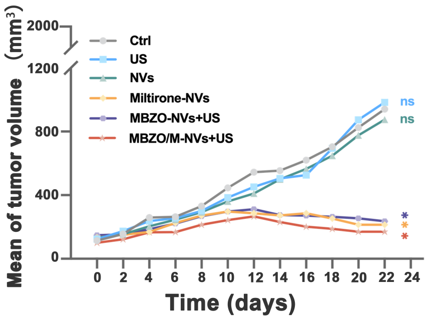

Fig. S21. Tumor growth curve after different treatments. Data are presented as mean ± SD (*n* = 6). Statistical analysis was determined using one-way ANOVA followed by Tukey's HSD (**P* < 0.05; ***P* < 0.01; ****P* < 0.001; n.s., not significance)

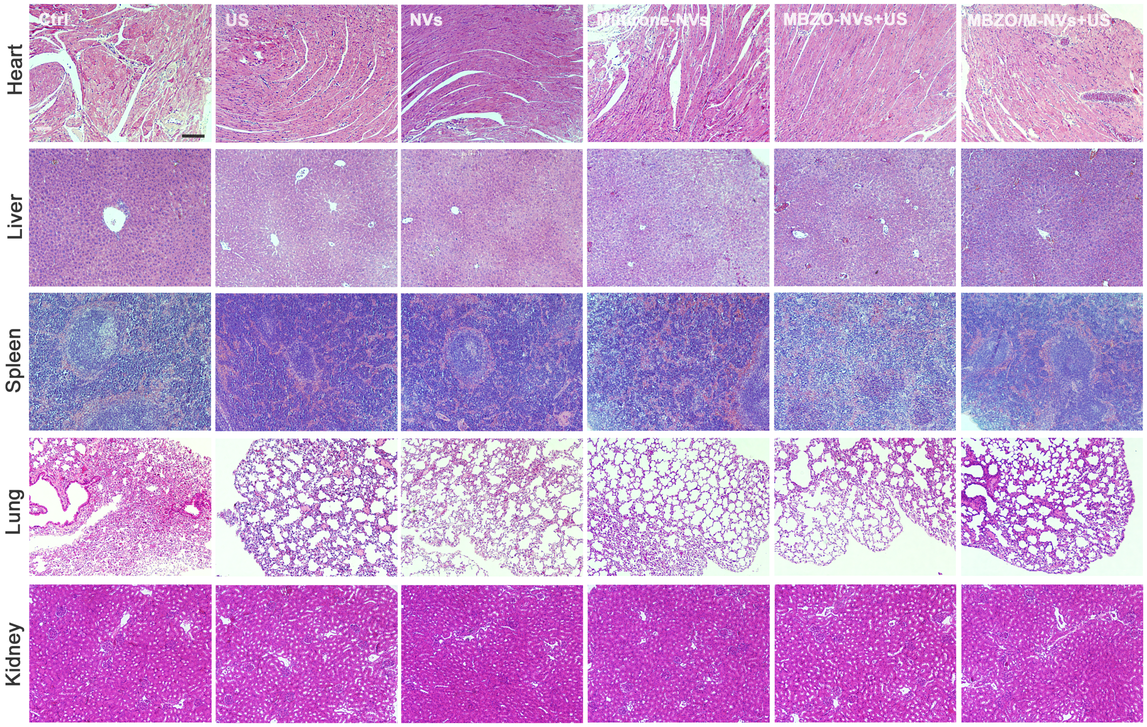

Fig. S22. H&E staining of the heart, liver, spleen, lung, and kidney tissues from mice in US, NVs, Miltirone-NVs, MBZO-NVs+US, and MBZO/M-NVs+US groups (Scalebar = 150 μm).

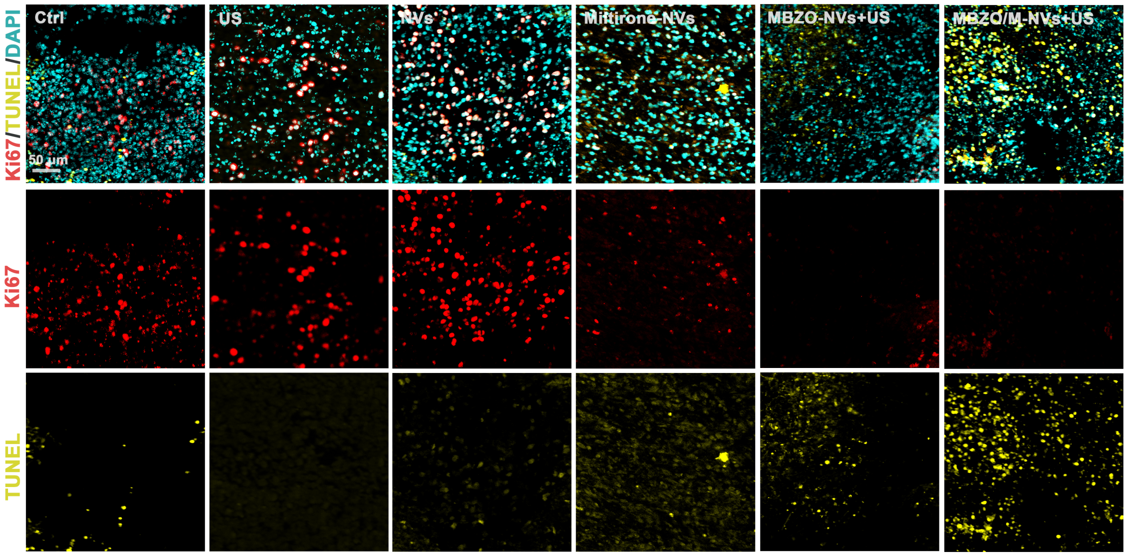

Fig. S23. Ki67/TUNEL staining of the tumor tissues receiving various treatments.

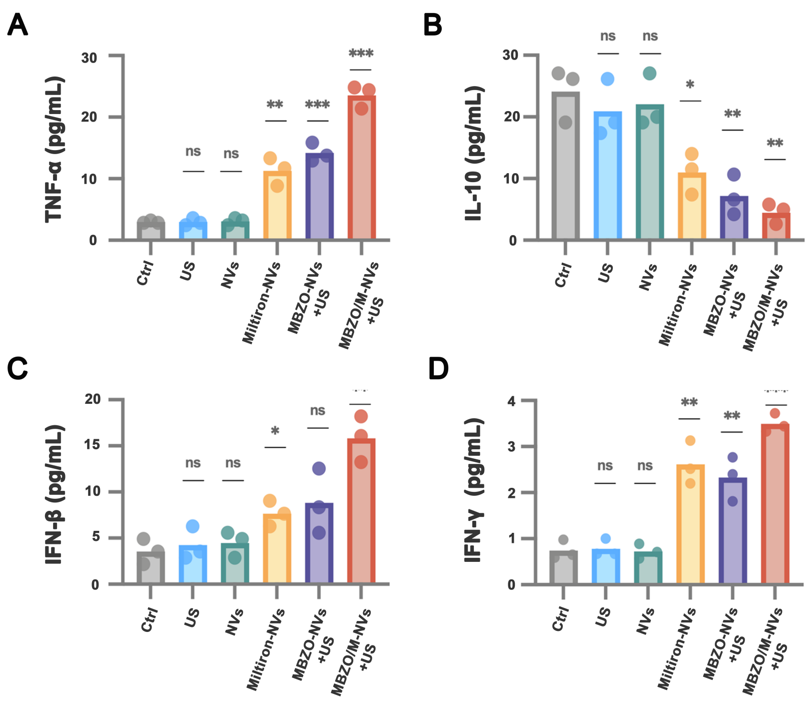

Fig. S24. (A-D) Cytokine levels of **(A)** TNF-α, **(B)** IL-10, **(C)** IFN-β, and **(D)** IFN-γ in the serum collected from mice after treatments. Data are presented as mean ± SD (n = 3). Statistical analysis was determined using one-way ANOVA followed by Tukey's HSD (*$P < 0.05$; **$P < 0.01$; ***$P < 0.001$; n.s., no significance).

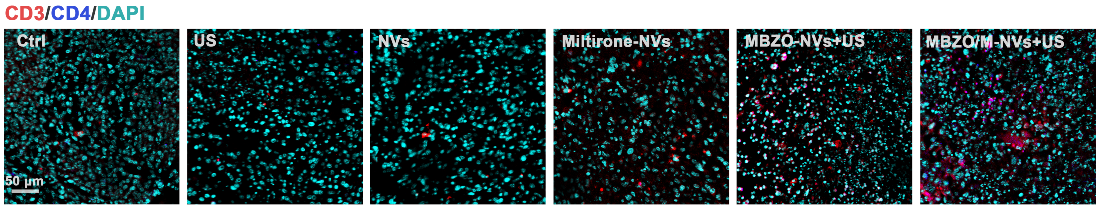

Fig. S25. Immunofluorescence staining of $CD3^{+}$ $CD4^{+}$ T cells infiltration in distant tumors of mice receiving various treatments.

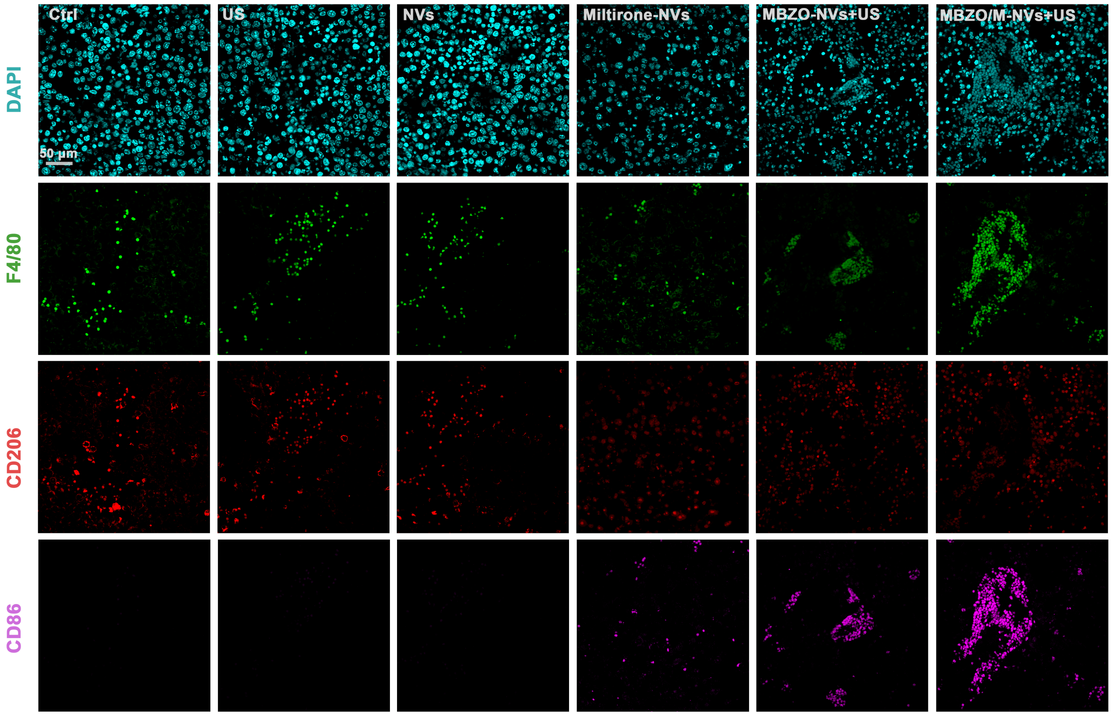

Fig S26. Single signal channel of immunofluorescence staining images to explore the F4/80$^+$ macrophage, including M1 macrophage (CD86$^+$) or M2 macrophage (CD206$^+$) infiltration in distant tumors of mice receiving various treatments.

Table S1. ICP-MS results of BZO, 1% MBZO, 2% MBZO, and 5% MBZO nanocubes.

Sample	Element	Mass concentration ($mg\ mL^{-1}$)	Molar mass fraction of cations	Mn doping ratio
BZO	Mn	0	50.17%	0
	Ba	14.33	49.83%	
	Zr	9.431	0	
1% MBZO	Mn	0.1015	0.85%	1.68%
	Ba	14.85	51.18%	
	Zr	9.246	47.97%	
2% MBZO	Mn	0.1238	1.04%	2.01%
	Ba	15.08	49.82%	
	Zr	9.873	49.14%	
5% MBZO	Mn	0.3514	2.71%	5.14%
	Ba	16.23	50.13%	
	Zr	10.14	47.16%	

Table S2. Primers used in qRT-PCR.

Primer (qRT-PCR)	Forward	Reverse
IFNA1	CTGGGAGGTTGTCAGAGCAG	GCAGGGGTGAGAGTCTTTGAA
IFNB1	AGTAGGCGACACTGTTCGTG	AGCCTCCCATTCAATTGCCA
MX1	GTTTCCGAAGTGGACATCGCA	GTTTCCGAAGTGGACATCGCA

Supplementary Materials and Methods

Evaluation of ROS generation

The thermoelectric current of BZO and MBZO nanocubes under temperature variations was measured by an electrochemical analyzer in the Na_2SO_4 aqueous solution (0.5 M) using a Pt plate as the counter electrode, and Ag/AgCl as a reference electrode, respectively. The working electrode was fabricated by depositing the suspension prepared from MBZO nanocubes (10 mg) mixed with Nafion (1.0 mM, 2 mL) in ethanol onto an FTO glass.

TMB was employed to detect the production of ·OH by nanocubes. Typically, nanocubes (20 μg mL^{-1}) were treated with or without US in 2 mL PBS solution (pH 5.5) containing TMB (0.5 mM). After co-incubating for 15 minutes, the absorbance of TMB at 652 nm was monitored to detect the generation of ·OH. In addition, the DTNB probe confirms the consumption of GSH. UV–vis absorption spectra of DTNB in MBZO nanocubes solutions (20 μg mL^{-1}) for different US irradiation times (0, 1, 3, and 5 minutes) with H_2O_2. DTNB mixed with H_2O_2 was also detected as a control group.

Quantitative analysis of ·OH production was performed on an ESR spectrometer set up with a magnetic field from 3347 to 3427 G, using 1.0 mm quartz tubes. TMB was used to detect ·OH generation. MBZO nanocubes (20 μg) and 10 μL TMB (1.0 M) were mixed in 1 mL of PBS with 0.5 mM H_2O_2. For comparison, TMB mixed with H_2O_2 was also detected.

Sonothermal Performance Measurements

To reveal the influence of the concentration of nanocubes on the sonothermal performance, different BZO and MBZO nanocube solutions (0, 100, 150, 200, and 250 μg mL^{-1}) were subjected to continuous US irradiation. The temperature was measured every 3 seconds using an IR thermal camera. Subsequently, the temperature of the BZO and MBZO nanocubes dispersion irradiated by the US at different concentrations (100 and 250 μg mL^{-1}) was recorded. Additionally, the stability of the sonothermal properties of BZO and MBZO nanocubes was evaluae stability of the sonothermal properties of BZO and MBZO nanocubes was evaluated. The heating and cooling temperatures of BZO and MBZO nanocube aqueous solutions under US irradiation for 96 seconds, followed by natural cooling to 25 °C (with the US stopped), were recorded for three cycles.

Specific Uptake

Huh7 cells were cultured in confocal culture dishes overnight for adherence. Subsequently, PBS solution (50 μL) containing MBZO/M-NVs (membrane protein content: 100 μg mL^{-1}) was added to the medium. Nanocubes and NVs were labeled with FITC and DiI, respectively. After incubation for 4 hours, Huh7 cells were stained with DIO for 25 minutes. Then, the cells were fixed with 4% paraformaldehyde (PFA) and stained with DAPI. Then, the cellular uptake of nanoparticles was analyzed using CLSM.

Isolation of mouse-derived myocardial fibroblast cells

Primary cardiomyocytes were isolated from 5-10 neonatal C57BL/6N mice (less than 7 days old). Hearts were excised, minced, and subjected to sequential digestion with 0.08% trypsin and 0.1% collagenase II at 37 °C. The digested tissue was pooled in cold complete medium, centrifuged, and resuspended. Cells were cultured in DMEM with 10% FBS and 2% antibiotics. After 2 hours, adherent cells were maintained with medium changes and used for co-cultured experiments.

In vitro cytotoxicity assays

Cytotoxicity evaluations of nanoparticles were performed on Huh-7 cells and HUVEC cells *via* the CCK-8 reagent. Huh-7 cells and HUVEC cells were seeded into separate 96-well plates. After 12 hours of incubation, the medium was removed and replaced with fresh medium containing different concentrations of nanoparticles (membrane protein content: 100, 50, 20, 10, 5, and 0 μg

mL^{-1}). After 4 hours of incubation, the cells of the US treatment groups were irradiated with US (1.0 MHz, 1.0 W cm^{-2}, 50% duty cycle) for 3 minutes and then cultured for an additional 20 hours. Then, the medium was discarded, and the fresh medium with CCK-8 reagent was added to each well. Finally, the absorbance values were measured at 450 nm using a microplate reader.

Fluorescence images

Huh7 cells were seeded into 12-well dishes and cultured overnight. Then, the cells were treated with different formulations: PBS, US, NVs, Miltirone-NVs, MBZO-NVs, and MBZO/M-NVs. After 4 hours of incubation, the cells of the US treatment groups were irradiated with US (1.0 MHz, 1.0 W cm^{-2}, 50% duty cycle) for 3 minutes and then cultured for an additional 20 hours.

For live/dead cell staining, after Huh7 cells were treated with the above treatments, the treated cells were stained with Calcein-AM (2 μM) and PI (4 μM) for 15 minutes. After fixing the cells with 4% PFA and washing them with PBS, all fluorescence images were acquired by CLSM.

The mitochondrial membrane potential, intracellular ROS, and O_2 levels were determined by JC-1, DCFH-DA, and $(Ru(dpp)_3)^{2+}Cl_2$ staining, respectively. The original medium was replaced and stained with probes at 37 °C for 30 minutes. Finally, the cells were washed three times with PBS for analysis. The cell fluorescence images were obtained using CLSM.

For immunofluorescence staining, Huh-7 cells were treated with nanoparticles or tissue sections, followed by fixation in 4% PFA or ice methanol, and then permeabilized with 0.3% Triton X-100. Nonspecific binding was blocked using 5% bovine serum albumin (BSA). The cells were then incubated overnight at 4 °C with primary antibodies. After washing three times with PBS, the corresponding secondary antibody was added and incubated for 1 hour. Finally, the cell nucleus was stained with DAPI and examined using CLSM.

Apoptosis assay

Huh7 cells were placed into 6-well dishes and cultured overnight. Then, the cells were treated with different formulations: PBS, US, NVs, Miltirone-NVs, MBZO-NVs, and MBZO/M-NVs. After 4 hours of incubation, the cells of the US treatment groups were irradiated with US (1.0 MHz, 1.0 W cm^{-2}, 50% duty cycle) for 3 minutes and then cultured for an additional 20 hours. All treated cells were trypsinized, washed, and quantified by an annexin V-FITC/PI apoptosis detection kit using a flow cytometer.

Western blot assay

Huh7 cells were seeded into 6-well dishes and allowed to reach 70% density. The cells were treated with different formulations: PBS, US, NVs, Miltirone-NVs, MBZO-NVs, and MBZO/M-NVs. After 4 hours of incubation, the cells of the US treatment groups were irradiated with US (1.0 MHz, 1.0 W cm^{-2}, 50% duty cycle) for 3 minutes and then cultured for an additional 20 hours. Subsequently, the cells were collected, lysed, and extracted. The protein concentration was quantified using the BCA method. Then, Western blotting was performed to determine the expression of marker proteins related to TME, PANoptosis, and the STING pathway.

RNA Sequencing

Huh7 cells treated with PBS or MBZO/M-NVs+US irradiation were collected, and total RNA from the tumor cells was extracted using TRIzol. For bulk RNA-seq, total RNA was sequenced on Illumina Novaseq 6000. Differentially expressed genes were identified with log2-fold change > 1.0 and false discovery rate (FDR) < 0.05.

RNA extraction and qRT-PCR analysis

Total RNA was isolated using the TRIzol reagent according to the instructions of the manufacturer. 500 ng of total RNA was reverse-transcribed into cDNA in a total reaction volume of 20 μL reaction volume using HiScript III RT SuperMix for qPCR according to the instructions. Then,

quantitative real-time PCR was performed in the reaction volume system containing diluted cDNA using SYBR green Premix EX Taq II on the ABI7500 PCR system.

ELISA assay

The ELISA procedure was performed following the manufacturer's instructions for each specific kit. Key steps included: coating with capture antibody (optional for pre-coated plates); blocking with blocking buffer at 37 °C for 1 to 2 hours; sample and standard incubation at 37 °C, 1 to 2 hours; incubation with biotinylated detection antibody and enzyme conjugate, and color development using TMB substrate, terminated with 2 M sulfuric acid. Absorbance was measured at 450 nm within 10 minutes after stopping the reaction.

In vitro DC activation

DC cell activation was performed using the transwell system (pore size 0.4 μm). Bone marrow-derived dendritic cells were isolated from C57BL/6N mice and cultured for 6 days with 50 ng/mL IL-4 and 10 ng/mL GM-CSF. Then, BMDC cells were seeded at a density of 1×10^5 cells per well in the upper chamber of the transwell system. Huh7 cells were seeded in the lower chamber with various treatments for 24 hours. Afterward, the DC cells from the upper chamber were harvested and labeled using antibodies, including APC anti-mouse CD11c antibody, FITC anti-mouse CD80 antibody, and PE anti-mouse CD86.

In vitro and in vivo CT/MR imaging performance

MBZO/M-NPs were dissolved in a PBS solution with varying concentrations (0, 1.25, 2.5, 5, 10, 15, and 20 mg mL^{-1}). CT imaging was performed at various sample concentrations. In vivo, CT and T_1-weighted MR imaging experiments were performed by intravenously injecting the MBZO/M-NVs solution (100 μL) at a concentration of 5 mg kg^{-1}. Then, the CT and T_1-weighted MR images were obtained before and after 1, 3, 6, 12, and 24 hours of injection.
